# Comparative Genomics Unveils the Habitat Adaptation and Metabolic Profiles of *Clostridium* in an Artificial Ecosystem for Liquor Production

**DOI:** 10.1128/msystems.00297-22

**Published:** 2022-05-02

**Authors:** Guan-Yu Fang, Li-Juan Chai, Xiao-Zhong Zhong, Zhen-Ming Lu, Xiao-Juan Zhang, Lin-Huan Wu, Song-Tao Wang, Cai-Hong Shen, Jin-Song Shi, Zheng-Hong Xu

**Affiliations:** a Key Laboratory of Industrial Biotechnology of Ministry of Education, School of Biotechnology, Jiangnan University, Wuxi, People’s Republic of China; b National Engineering Research Center for Cereal Fermentation and Food Biomanufacturing, Jiangnan University, Wuxi, People’s Republic of China; c School of Life Science and Health Engineering, Jiangnan University, Wuxi, People’s Republic of China; d Jiangsu Provincial Engineering Research Center for Bioactive Product Processing, Jiangnan University, Wuxi, People’s Republic of China; e Institute of Microbiology, Chinese Academy of Sciences, Beijing, People’s Republic of China; f National Engineering Research Center of Solid-State Brewing, Luzhou, People’s Republic of China; University of Delhi

**Keywords:** *Clostridium*, pit mud, CAZymes, horizontal gene transfer, short-chain fatty acids

## Abstract

*Clostridium* inhabiting pit mud (PM) is one of the important bacterial populations for synthesizing flavor compounds of Chinese strong-flavor baijiu. The long-term cereal fermentation with sorghum as the main raw material creates an environment rich in starch, ethanol, and organic acids (mainly lactic acid). However, the genetic factors underpinning *Clostridium*’s adaptation to PM remain poorly understood. Here, we performed comparative genomic analysis between 30 pit mud-associated (PMA) and 100 non-pit mud-associated (NPMA) *Clostridium* strains. Comparison analysis of the enrichment of KEGG pathways between PMA and NPMA *Clostridium* strains showed two-component system, flagellar assembly, and bacterial chemotaxis pathways related to environmental adaptation were enriched in PMA strains. The number of genes encoding alcohol dehydrogenase and l-lactate dehydrogenase in PMA *Clostridium* strains was significantly higher than that in NPMA, which is helpful for them to adapt to the ethanol- and lactic acid-rich environment. The analysis of carbohydrate-active enzymes demonstrated that glycoside hydrolases (GHs) was the most abundant family in all *Clostridium* strains, and genes encoding GH4 and GH13, involved in starch and sucrose metabolism, were enriched in PMA *Clostridium*. Horizontal gene transfer analysis revealed that multiple genes encoding the enzymes involved in carbohydrate and amino acid metabolism were transferred from *Bacillus* to *Clostridium* in pit mud. Most of the PMA *Clostridium* strains had good potential for butyric acid synthesis from ethanol, lactic acid, and starch. Collectively, this study furthers our understanding of the habitat adaptation and metabolic potential of PMA *Clostridium* strains.

**IMPORTANCE** Pit mud is a typical artificial ecosystem for Chinese liquor production. *Clostridium* inhabiting pit mud plays essential roles in the flavor formation of strong-flavor baijiu. The relative abundance of *Clostridium* increased with pit mud quality, further influencing the quality of baijiu. So far, the ecological adaptation of *Clostridium* to a pit mud-associated lifestyle is largely unknown. Here, comparative genomic analysis of pit mud-associated (PMA) and non-pit mud-associated (NPMA) *Clostridium* strains was performed. We found genes related to the metabolism of starch, ethanol, and lactic acid were enriched in PMA *Clostridium* strains, which facilitated their adaptation to the unique brewing environment. In addition, horizontal gene transfer contributed to the adaptation of *Clostridium* to pit mud. Our findings provide genetic insights on PMA *Clostridium* strains’ ecological adaptation and metabolic characteristics.

## INTRODUCTION

Strong-flavor baijiu is one of the popular and traditional Chinese liquors, and it accounts for above 70% of the total baijiu sales in China (1). Raw materials (mainly sorghum) are anaerobically fermented by multiple microorganisms in underground mud cellars for 2 to 6 months and then distilled to obtain strong-flavor baijiu. Through the comparative microbiome analysis of fermented grains and pit mud, it was found that microbial assembly inhabiting pit mud played essential roles in the formation of typical flavor compounds of strong-flavor baijiu, for example, fatty acids such as caproic acid and butyric acid (2, 3). Metagenomic analysis and biomass estimation by quantitative PCR demonstrated that pit mud microbiota was dominated by bacteria and archaea (4). Clostridia dominated the pit mud bacterial community, mainly scattered in the genera including *Caproiciproducens*, *Hydrogenispora*, *Syntrophomonas*, *Clostridium*, and *Sedimentibacter* (3–5). Previous work revealed that the relative abundance of *Clostridium* increased with pit mud quality, further influencing the quality of strong-flavor baijiu (5). One of the representative characteristics and uses of *Clostridium* species was the production of short- and medium-chain fatty acids such as acetic acid, butyric acid, and caproic acid (6). Metagenomic analysis and clone library analysis based on the key genes of butyrate synthesis showed that *Clostridium* species were the main potential butyric acid producers in pit mud (7). So far, among the clostridial strains isolated from pit mud, *Clostridium* species was the most diverse population, with various fatty acid metabolic characteristics (7–9). The important role of *Clostridium* in baijiu flavor formation attracted us to further study their functions in pit mud microbial ecosystem.

The genus *Clostridium* includes 130 species according to the EzBioCloud database (data collected in December 2021; https://www.ezbiocloud.net/), and these strains were isolated from a wide range of habitats, mainly including feces, soil, and foods (10). The living environment of *Clostridium* species inhabiting pit mud is closely related to human brewing activities. During the fermentation of strong-flavor baijiu, polymers in starch-rich raw materials (mainly sorghum) are hydrolyzed and metabolized into ethanol, organic acids, and other small molecules by microorganisms affiliated with *Lactobacillaceae*, *Kazachstania*, and *Wickerhamomyces* (3). These small molecules are dissolved in the water produced by the fermentation process, formed huangshui (the liquid produced by cereal fermentation), and gradually accumulated in the cellar under the action of gravity, so as to contact with the pit mud. Ethanol content in huangshui could reach about 7% to 10% (11). The accumulation of plenty of organic acids during baijiu fermentation created an acidic environment, and pH of fermented grains and huangshui was about 3 to 4 (3, 11). Lactic acid was the most abundant organic acid, and its content in fermented grains could reach ~30 g/kg at the end of fermentation (3). Lactic acid content in huangshui was around 40 g/liter or even 80 g/liter (11, 12). Thus, it is clear that the habitat environment of pit mud microorganisms is rich in organic acids (mainly lactic acid) and ethanol due to cereal fermentation. Interestingly, study on viable bacteria in pit mud of different ages revealed that the relative abundance of *Clostridium sensu stricto* was 12 to ~18% in 20-year-old cellar, significantly higher than that in 5-year-old cellar (0.4%) (13). How these *Clostridium* species survive and resist the environmental pressure of pit mud is not yet fully understood.

Comparative genomic analysis is an effective approach to illuminate the evolution and habitat adaptation mechanism of microorganisms (14–16) and the diverse metabolic capabilities of strains within the same species isolated from different habitats (17). Five Clostridium beijerinckii strains were isolated from pit mud, and comparative genomics of these strains and C. beijerinckii from other habitats showed that hundreds of genes were shared only by strains isolated from pit mud (18). To clarify the adaptation mechanism of *Clostridium* in pit mud environment, comparative genomics of 130 *Clostridium* strains from different habitats were performed in this study, including genome sequences of 30 strains isolated from pit mud and 100 strains from other habitats. Pangenome analysis was conducted to reveal the functional differences of core genome of pit mud-associated (PMA) and non-pit mud-associated (NPMA) *Clostridium* strains. Genes significantly enriched in PMA strains were identified to explain their adaptation to pit mud environment, and the effect of horizontal gene transfer on these enriched genes was evaluated. Considering the roles of *Clostridium* in the flavor formation of strong-flavor baijiu, we further compared the metabolic potentials of short-chain fatty acids between PMA and NPMA strains. This work provides insights into the genomic features of *Clostridium* species under the unique environment of pit mud.

## RESULTS

### Genome characteristics and phylogenetic composition of *Clostridium* strains.

**FIG 1:**
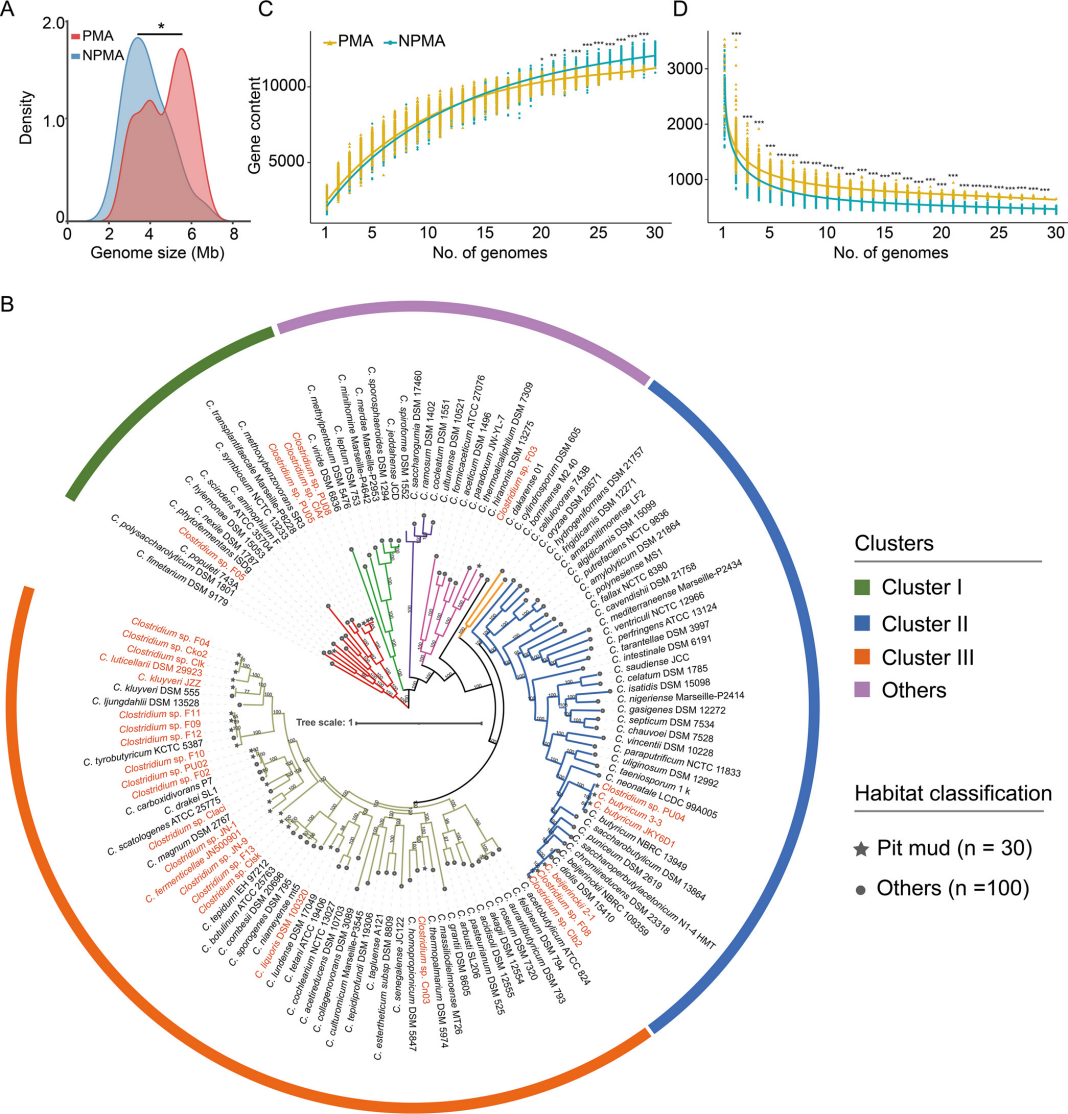
Comparative genomics and phylogenomics of *Clostridium* strains. (A) Genome size comparison between PMA and NPMA *Clostridium* strains. (B) The phylogenetic tree based on the protein sequences of single-copy genes of all *Clostridium* genomes using Fasttree under an approximate maximum-likelihood method. The names of *Clostridium* isolated from pit mud are highlighted in orange. The pan (C)- and core (D) genome sizes of PMA and NPMA *Clostridium* strains when randomly sampled with the same number of genomes are shown (Mann-Whitney *U* test, *, *P < *0.05; **, *P < *0.01; ***, *P < *0.001).

To decipher the underlying mechanism of the adaptation to pit mud environment of *Clostridium*, comparative genomic analysis was carried out between 30 pit mud-associated (PMA) and 100 non-pit mud-associated (NPMA) *Clostridium* strains (see Table S1 in the supplemental material). The 30 PMA strains were isolated from the pit mud of different fermentation cellars from strong-flavor baijiu producers in China. It should be noted that no matter what producer, the brewing process with sorghum as the main raw material creates a similar ecological environment for microorganisms inhabiting pit mud. To investigate the genomic differences between PMA and NPMA *Clostridium* strains, all of the genomes of *Clostridium* type strains isolated from other habitats and deposited in the EzBioCloud database were used for comparative genomics analysis. The genome sizes of PMA *Clostridium* strains were 4.53 ± 1.22 Mb on average, which was significantly larger than that of NPMA strains (3.92 ± 1.08 Mb) (Mann-Whitney *U* test, *P = *0.02) (Fig. 1A). The mean value of GC contents of PMA *Clostridium* strains (32% ± 7%) was significantly lower than that of NPMA strains (33% ± 5%) (Mann-Whitney *U* test, *P = *0.01) (Fig. S1).

10.1128/msystems.00297-22.1 FIG S1 Density plot of GC content of the genomes of pit mud-associated (PMA) and non-pit mud-associated (NPMA) *Clostridium* strains. Download FIG S1, PDF file, 0.4 MB. Copyright © 2022 Fang et al. 2022 Fang et al. https://creativecommons.org/licenses/by/4.0/ This content is distributed under the terms of the Creative Commons Attribution 4.0 International license.

10.1128/msystems.00297-22.6 TABLE S1 Genome statistics and project information of the *Clostridium* strains downloaded from NCBI and EzBioCloud databases. Download Table S1, DOCX file, 0.04 MB. Copyright © 2022 Fang et al. 2022 Fang et al. https://creativecommons.org/licenses/by/4.0/ This content is distributed under the terms of the Creative Commons Attribution 4.0 International license.

Core genome-based phylogenetic analysis of 130 *Clostridium* strains from different habitats was conducted to examine their evolutionary relationships. The maximum-likelihood phylogenetic tree was constructed based on the amino acid sequences of 304 single-copy core genes (Fig. 1B). PMA *Clostridium* strains fell into different clades, and 63.3% of them were scattered in cluster III. A previous study found that the distribution of microorganisms in the phylogenetic tree was not necessarily related to their habitats (19). The size of pan- and core genomes will be influenced by the number of genomes. Therefore, we randomly sampled the same number of genomes from PMA and NPMA *Clostridium* strains for pangenome analysis. When more than 19 genomes were sampled, the sizes of the pangenomes of NPMA *Clostridium* strains were significantly larger than those of PMA strains (Fig. 1C). When the number of sampled genomes was greater than one, the core genome sizes of PMA *Clostridium* strains were significantly larger than those of NPMA strains (Fig. 1D).

**FIG 2:**
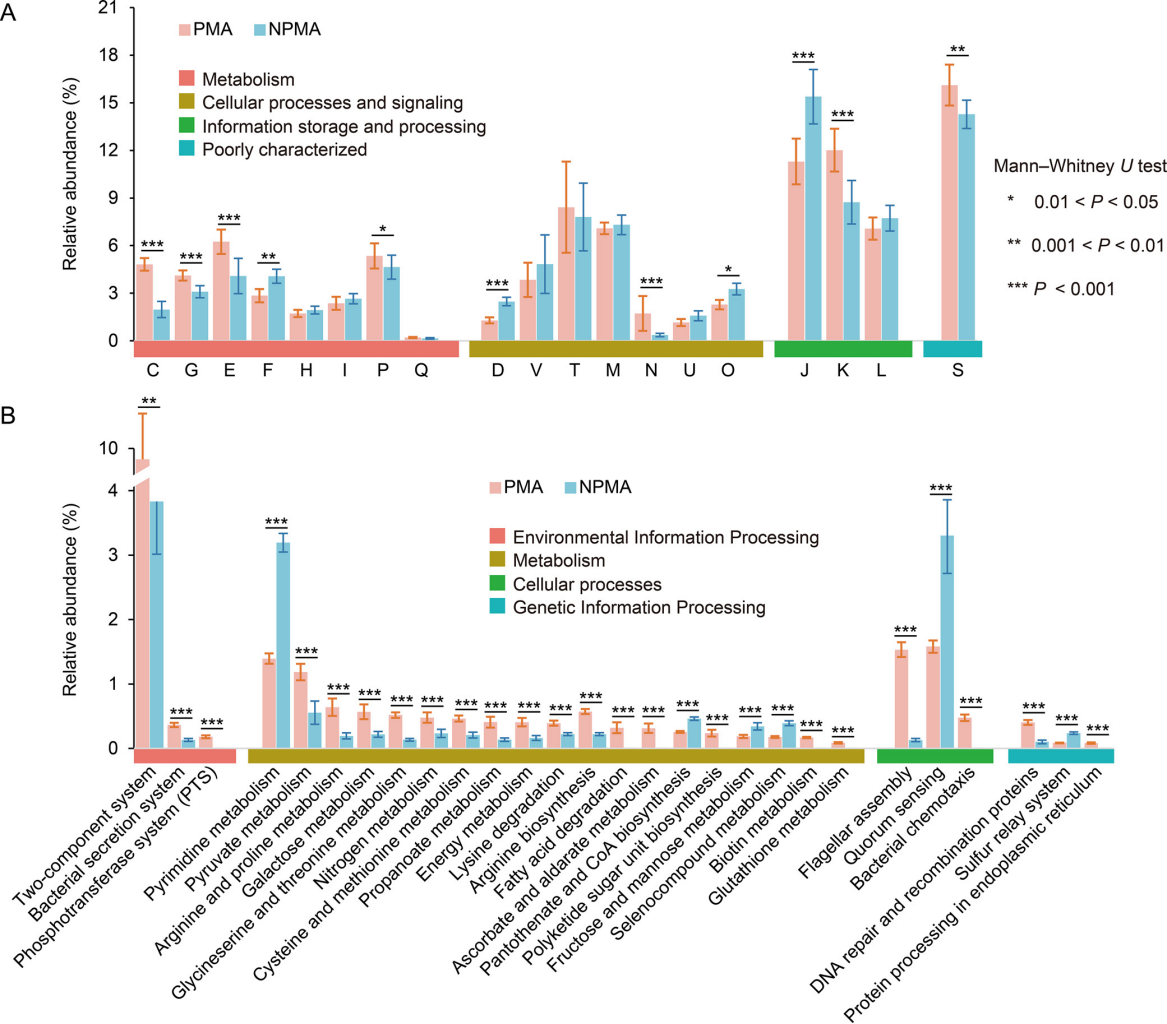
Functional categories of the core genome of pit mud-associated (PMA) and non-pit mud-associated (NPMA) *Clostridium* strains based on COG (A) and KEGG (B) databases (Mann-Whitney *U* test, *, *P < *0.05; **, *P < *0.01; ***, *P < *0.001). Bar charts represent the number of gene categories in statistical tests, and the error bars represent the standard deviations of the number of genes in the core genomes of PMA/NPMA *Clostridium* strains.

The comparison of the core genome functions between PMA and NPMA *Clostridium* strains was performed according to the methods of a previous study (20). A random set of 30 genomes of NPMA *Clostridium* strains was selected, and the mean phylogenetic distance between PMA and NPMA strains was calculated. This step was repeated 100 times, and then 30 genomes of NPMA strains with the least deviation from the average phylogenetic distance of PMA strains were selected for the subsequent analysis. An alignment to COG and KEGG databases was performed to analyze the similarities and differences in potential functions of the core genomes of PMA and NPMA *Clostridium* strains. The results based on COG database showed that the abundant enriched function of the core genome of all *Clostridium* strains included transcription (K), translation, ribosomal structure and biogenesis (J), signal transduction mechanisms (T), cell wall/membrane/envelope biogenesis (M), and replication, recombination, and repair (L) functional categories, accounting for more than 60% of the total abundance (Fig. 2A). The relative abundance of the COG categories involved in metabolism of energy production and conversion (C, 4.1% ± 0.3%), carbohydrate transport and metabolism (G, 4.2% ± 0.3%), amino acid transport and metabolism (E, 6.2% ± 0.7%), and inorganic ion transport and metabolism (P, 5.3% ± 0.8%) was significantly higher than that of NPMA strains (1.9% ± 0.5%, 3.1% ± 0.3%, 4.1% ± 1.1%, and 4.6% ± 0.8%), while the relative abundances of nucleotide transport and metabolism (F, 2.8% ± 0.4%) were lower than that of NPMA (4.1% ± 0.4%). As for the functional distribution of core genes based on KEGG database, the pathways were defined as significantly enriched in PMA/NPMA *Clostridium* strains if the *P* value in the Mann-Whitney *U* test was less than 0.05 and the difference in the number of genes in the pathway between PMA and NPMA strains was more than two times. The results showed that the pathways related to environmental adaptation, including two-component system, flagellar assembly, and bacterial chemotaxis, were significantly enriched in PMA *Clostridium* strains (Fig. 2B). A large number of genes related to carbohydrate metabolism (pyruvate metabolism, galactose metabolism, propanoate metabolism, and ascorbate and aldarate metabolism) and amino acid metabolism (arginine and proline metabolism, glycine-serine and threonine metabolism, cysteine and methionine metabolism, lysine degradation, and arginine biosynthesis) were significantly enriched in PMA *Clostridium* strains, while the pathways of pyrimidine metabolism, pantothenate and coenzyme A (CoA) biosynthesis, fructose and mannose metabolism, selenocompound metabolism, quorum sensing, and sulfur relay system were significantly enriched in NPMA *Clostridium* strains.

### Functional enrichment of *Clostridium* in different habitats.

**FIG 3:**
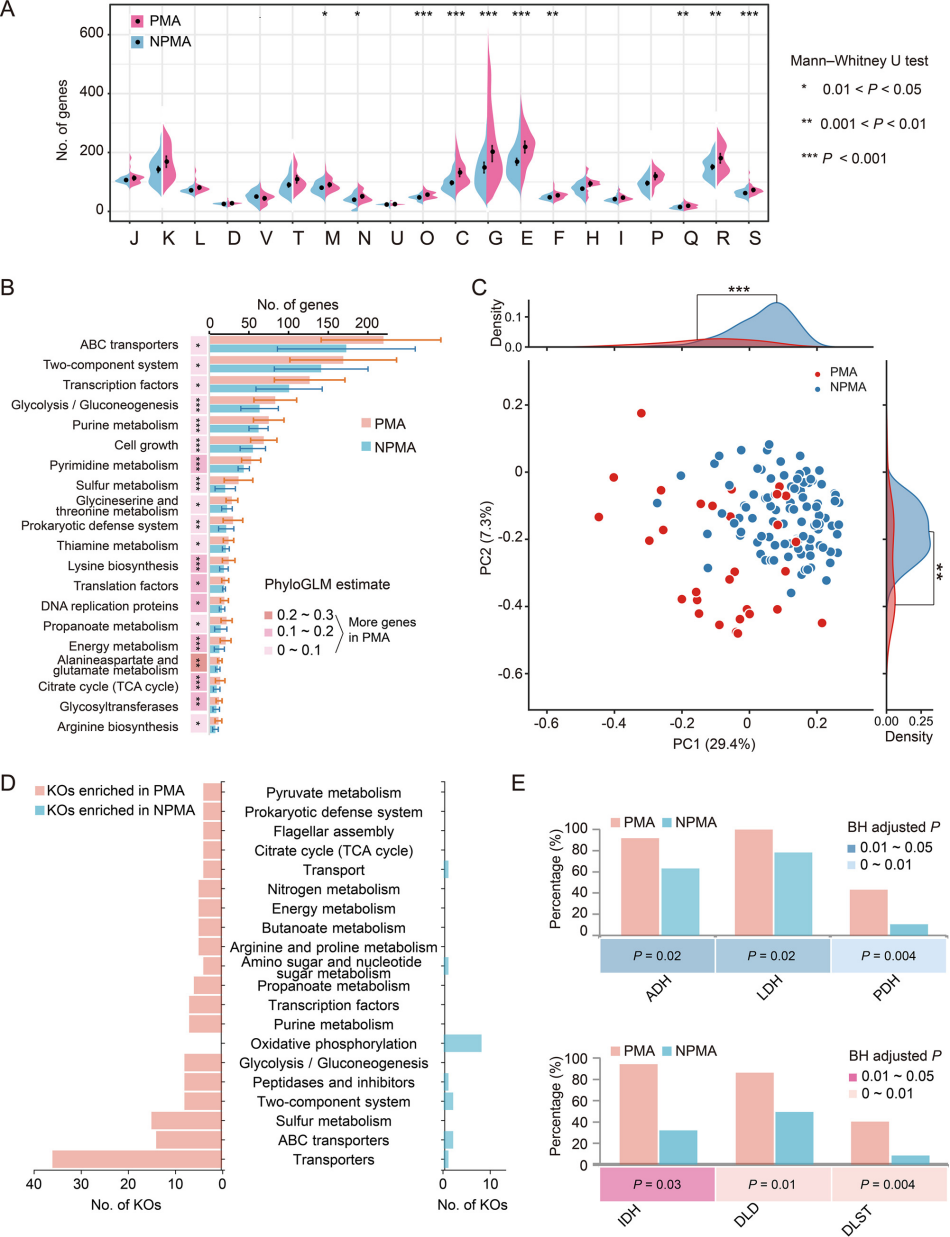
Enrichment analysis of functional categories in pit mud-associated (PMA) and non-pit mud-associated (NPMA) *Clostridium*. (A) Pairwise functional comparison among PMA and NPMA *Clostridium* strain genomes using COG categories. The boxes in the plot represent the gene copy number of COG categories (Mann-Whitney *U* test, *, *P < *0.05; **, *P < *0.01; ***, *P < *0.001). (B) The main KEGG pathways with significant differences in the number of genes (PhyloGLM test, *P < *0.05, estimate > 0). Pink color (PhyloGLM estimate > 0) indicates more genes in the genomes of PMA *Clostridium* strains than NPMA strains, while blue color (PhyloGLM estimate < 0) indicates more genes are enriched in NPMA strains. PhyloGLM test, *, *P < *0.05; **, *P < *0.01; ***, *P < *0.001. Bar charts represent the number of genes in this pathway in statistical tests, and the error bars represent the standard deviations of the number of genes in PMA/NPMA *Clostridium* strains. (C) Phylogenetically informed principal-component analysis (phylo-PCA) of the pathways with significant differences in gene content between PMA and NPMA *Clostridium* strains. (D) The 310 KOs with significant differences in the number of genes between PMA and NPMA *Clostridium* strains obtained in a stringent version of Scoary, a gene presence/absence approach that combines Fisher’s exact test, a phylogenetic test, and a label permutation test. (E) Comparison of the number of genes encoding alcohol dehydrogenase (ADH), lactate dehydrogenase (LDH), pyruvate dehydrogenase (PDH), isocitrate dehydrogenase (IDH), dihydrolipoamide dehydrogenase (DLD), and 2-oxoglutarate dehydrogenase (DLST).

Previous studies have shown that the genomic functions of bacteria with close phylogenetic relationships are shaped by their habitats and lifestyles (21, 22). The differences of core genome functions between PMA and NPMA *Clostridium* strains were analyzed above. Here, a comparative analysis of the genome-wide functions enriched in PMA and NPMA *Clostridium* strains was conducted. The analysis based on gene copy numbers showed that more genes were significantly (Mann-Whitney *U* test, *P < *0.05) enriched in 10 COG categories of PMA *Clostridium* strains relative to NPMA strains, especially posttranslational modification, protein turnover, chaperones (O), energy production and conversion (C), carbohydrate transport and metabolism (G), and amino acid transport and metabolism (E) (*P < *0.001) (Fig. 3A). Moreover, the numbers of genes enriched in these four COG categories (O, C, G, and E) were also significantly (*P < *0.001) higher in PMA *Clostridium* strains than in NPMA strains based on presence/absence (Fig. S3A).

10.1128/msystems.00297-22.3 FIG S3 (A) Pairwise functional comparison among pit mud-associated (PMA) and non-pit mud-associated (NPMA) *Clostridium* strain genomes using COG categories based on a gene presence/absence dataset. (B) The main KEGG pathways with significant differences in the number of genes. (C) Comparison of the number of genes encoding alcohol dehydrogenase (ADH), lactate dehydrogenase (LDH), pyruvate dehydrogenase (PDH), isocitrate dehydrogenase (IDH), dihydrolipoamide dehydrogenase (DLD), and 2-oxoglutarate dehydrogenase (DLST). Bar charts represent the number of genes in statistical tests, and the error bars represent the standard deviations of the number of genes in PMA/NPMA *Clostridium* strains. *, *P < *0.05; **, *P < *0.01; ***, *P < *0.001, Mann-Whitney *U* test. Download FIG S3, PDF file, 1.1 MB. Copyright © 2022 Fang et al. 2022 Fang et al. https://creativecommons.org/licenses/by/4.0/ This content is distributed under the terms of the Creative Commons Attribution 4.0 International license.

Comparison analysis of the KEGG pathways between PMA and NPMA *Clostridium* strains was performed by PhyloGLM test and phylogenetically informed principal component analysis (phylo-PCA) (Fig. 3B and C). The results showed that the number of genes in 45 pathways was significantly different between PMA and NPMA *Clostridium* strains (PhyloGLM test, *P < *0.05) (Table S2), and the top 20 pathways are shown in Fig. 3B. Forty-three pathways were enriched in PMA strains, and many of these metabolic pathways were related to environmental adaptation, including two-component system (23), energy metabolism (24), and bacterial chemotaxis (25) pathways; however, only two pathways were enriched in NPMA strains. A large number of genes related to carbohydrate metabolism (glycolysis/gluconeogenesis, citrate cycle, glycosyltransferases, and propanoate metabolism) and amino acid metabolism (glycine-serine and threonine metabolism, lysine biosynthesis, alanine-aspartate and glutamate metabolism, arginine biosynthesis, tyrosine metabolism, and valine-leucine and isoleucine degradation) were enriched in PMA *Clostridium* (Fig. 3B). Phylo-PCA analysis showed that the enriched KEGG pathways in PMA and NPMA strains were divided into two clusters, which were significantly different (Mann-Whitney *U* test, *P < *0.01) on both the PC1 and PC2 axes, suggesting that these pathways are related to different habitat characteristics.

10.1128/msystems.00297-22.7 TABLE S2 The functional composition by level 3 of KEGG orthology classification with significant differences in the number of genes (PhyloGLM test, *P < *0.05). Download Table S2, DOCX file, 0.02 MB. Copyright © 2022 Fang et al. 2022 Fang et al. https://creativecommons.org/licenses/by/4.0/ This content is distributed under the terms of the Creative Commons Attribution 4.0 International license.

Moreover, the clade-based analysis was performed to investigate the differences of gene content between PMA and NPMA *Clostridium* strains on different clades of the phylogenetic tree. The results showed that the pathways of ABC transporters, two-component system, glycolysis/gluconeogenesis, cell growth, energy metabolism, and citrate cycle (tricarboxylic acid [TCA] cycle) were significantly enriched in PMA *Clostridium* in all clusters (Fig. S3B). A large number of genes related to amino acid metabolism were enriched in PMA strains in clusters II and III. The pathways of transcription factors, purine metabolism, and arginine biosynthesis were enriched in PMA strains in clusters I and II, and the prokaryotic defense system pathway was enriched in PMA strains in clusters I and III.

Three hundred and ten KEGG orthologs (KOs) with significant differences between PMA and NPMA *Clostridium* strains were obtained by pan-genome-wide association studies (pan-GWAS) based on the presence/absence data of KOs, of which 281 KOs were enriched in PMA *Clostridium*, and the other 29 KOs were enriched in NPMA *Clostridium* (Fig. 3D). Among the enriched KOs in PMA *Clostridium* strains, it could be observed that 36, 15, 14, 8, and 8 KOs belonged to transporters, sulfur metabolism, ABC transporters, two-component system, and glycolysis/gluconeogenesis, respectively, while for NPMA *Clostridium*, eight KOs belonging to oxidative phosphorylation, two KOs belonging to ABC transporters, and two KOs belonging to two-component system were enriched (Fig. 3D). Pit mud is exposed to the environment of grain fermentation for extended periods of time, and there were a large number of yeasts and lactic acid bacteria living in this environment (3). Due to the metabolism of these microorganisms, a large amount of lactic acid and ethanol will be accumulated in pit mud (12, 26). The stress of the environment with high concentrations of lactic acid and ethanol can inhibit the growth of microorganisms. Therefore, the genes of enzymes that can directly metabolize ethanol and lactic acid were analyzed. The number of genes encoding alcohol dehydrogenase (EC 1.1.1.1, K13954), oxidizing ethanol into acetaldehyde, in PMA *Clostridium* strains was significantly higher than that in NPMA *Clostridium* strains (Fisher’s exact test, Benjamini-Hochberg adjusted *P < *0.05) (Fig. 3E). Ninety percent of PMA *Clostridium* strains had the alcohol dehydrogenase-coding genes, while that of NPMA *Clostridium* strains was only 66.0%. The proportion of strains with the genes encoding l-lactate dehydrogenase (EC 1.1.1.27, K00016) in PMA *Clostridium* (100%) was significantly higher than that in NPMA (80.0%). l-Lactate dehydrogenase plays a role in metabolizing lactic acid into pyruvate. Multiple genes encoding enzymes in the citrate cycle were enriched in PMA *Clostridium*, including dihydrolipoamide dehydrogenase (EC 1.8.1.4, K00382), isocitrate dehydrogenase (EC 1.1.1.42, K00031), pyruvate dehydrogenase (EC 1.2.4.1, K00162), and 2-oxoglutarate dehydrogenase (EC 2.3.1.61, K00658) (Fig. 3E). These genes play critical roles in the degradation of isocitric acid, oxalosuccinic acid, succinic acid, and other organic acids (27). Therefore, the enrichment of these genes in PMA *Clostridium* strains may play an important role in adapting to the environment of pit mud rich in organic acids.

The results of phylogenetic tree clade-based analysis showed that there was no statistical difference in the content of these genes between PMA and NPMA *Clostridium* strains in cluster I (Fig. S3C). In cluster II, the number of genes encoding alcohol dehydrogenase (2.0 ± 0.0), l-lactate dehydrogenase (3.9 ± 0.2), and isocitrate dehydrogenase (1.0 ± 0.0) in PMA *Clostridium* strains was significantly higher than that of NPMA strains (1.3 ± 0.7, 1.9 ± 0.6, and 0.7 ± 0.2). In cluster III, the number of genes encoding alcohol dehydrogenase (0.9 ± 0.2), l-lactate dehydrogenase (1.0 ± 0.0), pyruvate dehydrogenase (1.0 ± 0.1), dihydrolipoamide dehydrogenase (0.7 ± 0.2), and 2-oxoglutarate dehydrogenase (0.6 ± 0.2) in PMA *Clostridium* strains was significantly higher than that of NPMA strains (0.7 ± 0.2, 0.8 ± 0.2, 0.7 ± 0.2, 0.2 ± 0.1, and 0.2 ± 0.1).

### CAZyme profiling.

**FIG 4:**
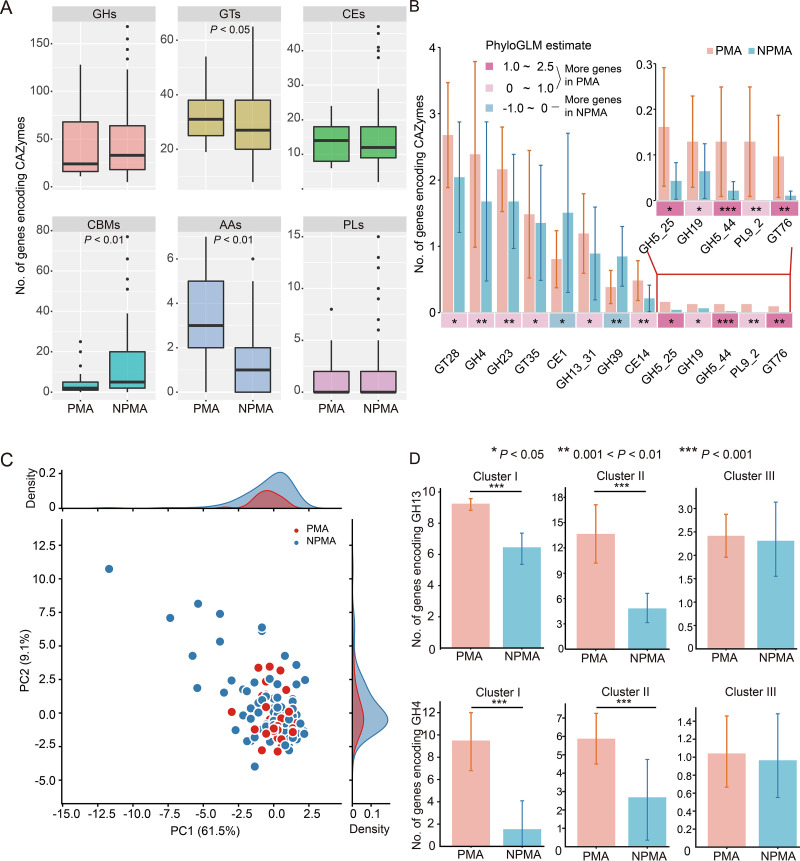
Carbohydrate-active enzymes (CAZymes) in all *Clostridium* strains. (A) Pairwise gene number of CAZyme comparisons among PMA and NPMA *Clostridium* strains. Box plot represents the diversity of CAZyme family content based on copy number data set (Mann-Whitney *U* test, *, *P < *0.05; **, *P < *0.01; ***, *P < *0.001) of auxiliary activities (AAs), carbohydrate-binding molecules (CBMs), carbohydrate esterases (CEs), glycoside hydrolases (GHs), glycosyltransferases (GTs), and polysaccharide lyases (PLs). (B) The significantly enriched CAZymes between PMA and NPMA *Clostridium* strains (PhyloGLM test, *P < *0.05). Pink color (PhyloGLM estimate > 0) indicates more genes in the genomes of PMA *Clostridium* strains than NPMA strains, while blue color (PhyloGLM estimate < 0) indicates more genes are enriched in NPMA strains. PhyloGLM test, *, *P < *0.05; **, *P < *0.01; ***, *P < *0.001. Bar charts represent the number of genes in this CAZyme family in statistical tests, and the error bars represent the standard deviations of the number of genes in PMA/NPMA *Clostridium* strains. (C) Phylogenetically informed principal component analysis (phylo-PCA) of the significantly enriched genes encoding CAZymes. (D) Comparison of the number of genes encoding GH4 and GH13 between PMA and NPMA *Clostridium* strains.

The above-described analysis showed that a large number of carbohydrate metabolism genes encoding enzymes in glycolysis/gluconeogenesis, citrate cycle, glycosyltransferases, and propanoate metabolism were enriched in PMA *Clostridium* strains (Fig. 3B and Table S2). Pit mud is in a carbohydrate-rich environment; therefore, carbohydrate-active enzymes (CAZymes) were analyzed using dbCAN2 to investigate the genomic potential for carbohydrate utilization of all *Clostridium* strains. There were 266 CAZyme families (13,590 genes) identified in all strains, including 145 glycoside hydrolases (GHs), 15 carbohydrate esterases (CEs), 24 polysaccharide lyases (PLs), 29 glycosyl transferases (GTs), 42 carbohydrate-binding protein module (CBM) families, and seven auxiliary activities (AAs). The number of genes encoding CAZymes varied greatly between *Clostridium* strains, ranging from 20 to 284 (Fig. 4A and Fig. S4A).

10.1128/msystems.00297-22.4 FIG S4 (A) Total number of genes encoding CAZymes in each strain distributed on six categories of enzyme activity: auxiliary activities (AAs), carbohydrate-binding molecules (CBMs), carbohydrate esterases (CEs), glycoside hydrolases (GHs), glycosyltransferases (GTs), and polysaccharide lyases (PLs). The number of genes encoding glycoside hydrolases (B) and glycosyltransferases (C) in cluster I, cluster II, and cluster III *Clostridium* strains is shown. *, *P < *0.05; **, *P < *0.01; ***, *P < *0.001, Mann-Whitney *U* test. Download FIG S4, PDF file, 1.5 MB. Copyright © 2022 Fang et al. 2022 Fang et al. https://creativecommons.org/licenses/by/4.0/ This content is distributed under the terms of the Creative Commons Attribution 4.0 International license.

GHs and GTs were the two abundant families of CAZymes in all *Clostridium* strains, with the number of genes encoding GHs and GTs accounting for 43.5% and 29.0%, respectively, of all CAZyme-coding genes (Fig. 4A and Fig. S4A). The GH superfamily is involved in glucan hydrolysis, including starch, cellulose, xylan, chitin, hemicellulose, and glycogen (28). The number of genes encoding GHs in PMA and NPMA *Clostridium* strains did not differ significantly. Accordingly, the number of genes encoding CAZymes between PMA and NPMA *Clostridium* strains was compared based on different clusters in the phylogenetic tree (Fig. 1B). The number of genes encoding GHs in PMA strains from cluster I (112.5 ± 31.0) was significantly higher than that of NPMA strains (64.1 ± 47.9), while there was no significant difference for strains on cluster II (PMA, 77.4 ± 15.6; NPMA, 58.0 ± 37.8) and III (PMA, 19.9 ± 7.1; NPMA, 27.6 ± 20.8) (Fig. S4B). GTs are essential for producing diverse and complex glycoconjugates (29). PMA *Clostridium* strains had significantly more genes encoding GTs than NPMA *Clostridium* strains (Fig. 4A). PMA strains on clusters I (31.3 ± 1.5) and II (39.1 ± 5.7) possessed significantly more genes encoding GTs than NPMA strains (cluster I, 26.1 ± 7.4; cluster II, 32.1 ± 10.6), while there was no significant difference between PMA (30.8 ± 9.8) and NPMA (29.2 ± 12.6) *Clostridium* strains on cluster III (Fig. S4C).

A PhyloGLM test was performed to detect the significantly different CAZymes between PMA and NPMA *Clostridium*. The results showed that the number of genes encoding 13 CAZymes varied significantly between PMA and NPMA *Clostridium* strains (PhyloGLM test, *P < *0.05). Eleven CAZymes (GT28, GH4, GH23, GT35, GH13_31, CE14, GH5_25, GH19, GH5_44, PL9_2, and GT26) were enriched in PMA *Clostridium*, and only two CAZymes (CE1 and GH39) were enriched in NPMA *Clostridium* (Fig. 4B). Phylo-PCA analysis showed the NPMA *Clostridium* strains were scattered on the first two principal components while the PMA *Clostridium* strains were aggregated (Fig. 4C), suggesting that the composition of the genes encoding CAZymes in PMA *Clostridium* were similar. The differences in CAZymes among NPMA *Clostridium* strains may be due to the wide range of their habitats.

GH4 and GH13 mainly included α-glucosidase, α-amylase, maltose-6-phosphate glucosidase, and maltogenic amylase, and these enzymes were involved in starch, oligosaccharide, and glycogen metabolic pathways (30). Sorghum is the main raw material for baijiu fermentation, providing a starch-rich environment for pit mud microorganisms. Therefore, the analysis of GH4- and GH13-coding genes was conducive to understanding the potential of *Clostridium* to metabolize carbohydrates. The abundant GH13 families included GH13_1, GH13_2, GH13_4, GH13_5, GH13_8, GH13_9, GH13_11 GH13_13, GH13_14, GH13_15, GH13_18, GH13_19, GH13_20, GH13_21, GH13_28, GH13_29, GH13_31, and GH13_36. On clusters I and II, the number of genes encoding GH13 in PMA *Clostridium* strains (cluster I, 9.25 ± 0.5; cluster II, 14.5 ± 4.4) was significantly higher than that in NPMA *Clostridium* strains (cluster I, 7.3 ± 3.4; cluster II, 7.1 ± 4.5), while there was no significant difference between PMA (2.4 ± 1.2) and NPMA (2.3 ± 2.3) *Clostridium* strains on cluster III (Fig. 4D). Similar to the genes encoding GH13, the number of genes encoding GH4 in PMA *Clostridium* strains (cluster I, 9.5 ± 5.0; cluster II, 5.9 ± 2.0) on clusters I and II was significantly higher than that in NPMA strains (cluster I, 1.5 ± 1.4; cluster II, 2.7 ± 2.3), and there was no significant difference between PMA and NPMA *Clostridium* strains on cluster III (Fig. 4D).

### Horizontal gene transfer in PMA *Clostridium* strains.

**FIG 5:**
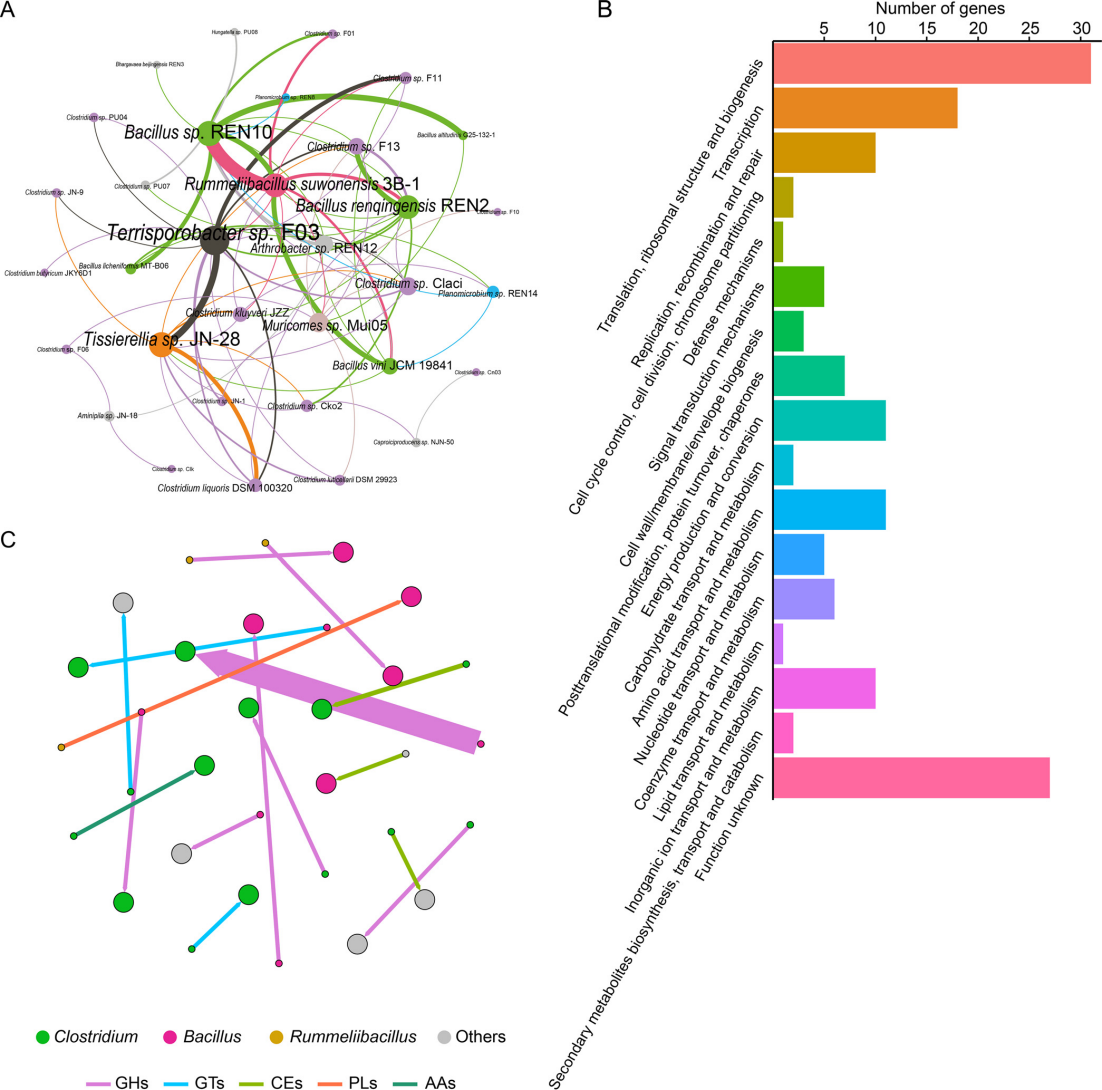
Horizontal gene transfer between pit mud bacteria. (A) Horizontal gene transfer among different strains of pit mud bacteria. The color of lines was the same as that for donors. (B) The functional categories of the horizontally transferred genes based on COG databases. (C) Transferred genes encoding CAZymes between pit mud bacteria.

Horizontal gene transfer (HGT) plays a crucial role in the environmental adaptation of bacteria. Bacteria can share resistance and metabolic genes through HGT under the stress of the environment (31, 32). The BLAST-based method was used to infer the HGT events among PMA strains to evaluate the roles of HGTs in pit mud adaptation of PMA bacteria. Fifty-six genomes of bacteria isolated from pit mud were used for HGT analysis, and the ecological environments of pit mud were similar due to the brewing process with sorghum as the main raw ingredient. These pure strains isolated from pit mud used for HGT analysis mainly affiliated with *Clostridia* and *Bacilli* at the class level and *Clostridium* and *Bacillus* at the genus level (Table S3), which attracted researchers’ attention because of their high abundance and their important functions in the flavor formation of strong-flavor baijiu (1, 3, 4). A total of 161 HGT events were detected in all PMA bacterial strains. These HGT events mainly occurred between the classes *Clostridia* and *Bacilli*, and the number of genes transferred from *Bacilli* to *Clostridia* was higher than that from *Clostridia* to *Bacilli* (Fig. S5A to D). At the genus level, there were a lot of HGT events that occurred between the genera *Clostridium* and *Bacillus* (Fig. 5A). Functional annotation of the horizontally transferred genes was performed by running similarity searches against the COG database (Fig. 5B). The results showed that the main functions of these horizontally transferred genes were translation, ribosomal structure and biogenesis (J), transcription (K), amino acid transport and metabolism (E), and energy production and conversion (C). The results of KEGG annotation showed that multiple genes encoding the enzymes involved in starch and sucrose metabolism pathway, including maltase-glucoamylase, maltose-6′-phosphate glucosidase, 6-phospho-beta-glucosidase, and sucrase-isomaltase, were transferred from *Bacillus* to *Clostridium* (Fig. S5F), and these enzymes play important roles in the metabolism of starch. The transferred genes encoding CAZymes were detected against the CAZy database (Fig. 5C). Genes encoding CAZymes were mainly transferred from *Bacilli* to *Clostridia* (Fig. S5E). Multiple genes encoding GH13 and GH135 were transferred from *Bacillus* to *Clostridium* (Fig. S5G). GH13 can be directly involved in the metabolism of glycogen and starch (30), which was enriched in PMA *Clostridium* strains (Fig. 4D). There were several genes encoding GHs transferred among *Clostridium* strains. Multiple genes encoding GTs, CEs, and AAs were transferred from other bacterial genera to *Clostridium* or among *Clostridium* strains.

10.1128/msystems.00297-22.5 FIG S5 Horizontally transferred genes among bacterial strains isolated from pit mud at class (A), order (B), family (C), and genus (D) levels. (E) Transferred genes encoding CAZymes between the classes *Bacilli* and *Clostridia*. (F) The genes transferred from *Bacillus* to *Clostridium* involved in starch and sucrose metabolism pathway. (G) The genes encoding GH13 and GH135 transferred from *Bacillus* to *Clostridium*. Download FIG S5, PDF file, 1.4 MB. Copyright © 2022 Fang et al. 2022 Fang et al. https://creativecommons.org/licenses/by/4.0/ This content is distributed under the terms of the Creative Commons Attribution 4.0 International license.

10.1128/msystems.00297-22.8 TABLE S3 Genome statistics and project information of the *Clostridium* strains downloaded from NCBI used for horizontal gene transfer. Download Table S3, DOCX file, 0.02 MB. Copyright © 2022 Fang et al. 2022 Fang et al. https://creativecommons.org/licenses/by/4.0/ This content is distributed under the terms of the Creative Commons Attribution 4.0 International license.

### Pathway analysis of the synthesis of short-chain fatty acids.

**FIG 6:**
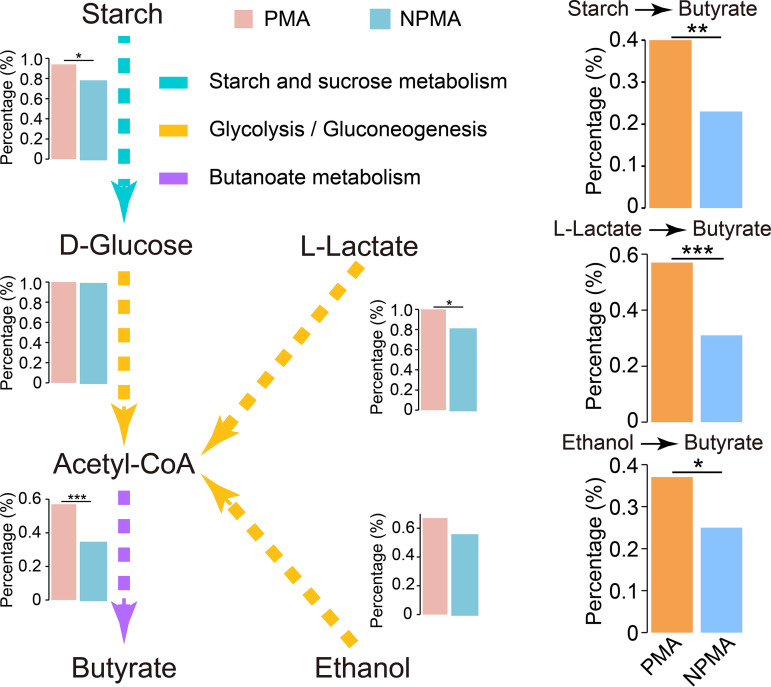
Metabolic pathways analysis of synthesis of butyrate from ethanol, lactic acid, and starch in *Clostridium* strains. The histogram represents the proportion of strains with these genes in this pathway, and we calculated a *P* value using the Poisson distribution (*, *P < *0.05; **, *P < *0.01; ***, *P < *0.001).

The pit mud is enriched with high concentrations of ethanol, lactic acid, and starch, which is significantly different from other habitats (3, 12). Ethanol, lactic acid, and starch are important precursors for the synthesis of short-chain fatty acids, which are the key flavor substances of baijiu (33). Therefore, the potential for synthesizing short-chain fatty acids from these substances was compared between PMA and NPMA *Clostridium* strains (Fig. 6). The proportion of PMA *Clostridium* strains (93.3%) containing complete metabolic pathways from starch to glucose was higher than that of NPMA *Clostridium* strains (77.0%). To determine significance of differences in metabolic pathways between PMA *Clostridium* strains and NPMA *Clostridium* strains, we calculated the expected number of strains with the complete pathways and the observed number of strains and calculated a *P* value using the Poisson distribution (ppois R function). Glucose can be metabolized into pyruvate through glycolysis/gluconeogenesis, and there was no significant difference in the number of strains with this pathway between PMA and NPMA *Clostridium* strains. Lactic acid can be oxidized to pyruvate by lactate dehydrogenase (EC 1.1.1.27), and pyruvate can be metabolized to acetyl-CoA. All of the strains have genes encoding the enzymes that convert pyruvate to acetyl-CoA, while the number of strains with the gene encoding lactate dehydrogenase was significantly different between PMA and NPMA *Clostridium* strains. The proportion of the PMA *Clostridium* strains (100%) with the genes encoding lactate dehydrogenase was significantly higher than that in NPMA *Clostridium* strains (80.0%) (Poisson distribution, *P < *0.05). There was no significant difference in the number of strains with complete metabolic pathway from ethanol to acetyl-CoA between PMA (66.7%) and NPMA (55.0%) *Clostridium* strains.

Butyrate can be synthesized from acetyl-CoA through butanoate metabolism, and the number of PMA *Clostridium* strains (56.7%) with this metabolic pathway was significantly higher than that of NPMA *Clostridium* strains (34.0%) (Poisson distribution, *P < *0.001). The complete pathways of butyrate synthesis from starch, lactic acid, and ethanol of all *Clostridium* strains then were detected to estimate the potential for synthesis of butyrate. The proportion of the PMA *Clostridium* strains (40.0%, 56.7%, and 36.7%, respectively) synthesizing butyrate from starch, lactic acid, and ethanol was significantly higher than that of the NPMA *Clostridium* strains (23.0%, 31.0%, and 25.0%, respectively) (Poisson distribution, *P < *0.05).

## DISCUSSION

Microorganisms will be filtered by the specific environments of their habitats, so only a part of them can survive in the specific habitat, which often leads to significant differences in the microbial genome features among the habitats (34–36). *Clostridium* strains were isolated from a range of habitats, as summarized in the EzBioCloud database (10). *Clostridium* was the predominant bacteria in pit mud during Chinese strong-flavor baijiu production, playing key roles in the production of short-chain fatty acids in baijiu (7). Due to the long-term cereal fermentation with sorghum as the main raw material, pit mud is enriched with plenty of organic acids, ethanol, and starch (3, 12). The genomic adaptation of *Clostridium* to the habitat of pit mud remains poorly understood. Here, a comparative genomics analysis was performed on the 130 *Clostridium* genomes to provide insights into the genomic features of *Clostridium* strains isolated from pit mud.

Bacteria that live in a nutrient-enriched environment tend to have larger genomes, and gene function is more redundant in these bacteria (37). It was found that the average genome size of PMA *Clostridium* strains was significantly larger than that of NPMA *Clostridium* strains. This may be a result of pit mud’s being rich in carbohydrates and amino acids, while the contents of nutrients of NPMA environments are relatively low compared with pit mud environment (10, 38). Bacteria with larger genomes accumulate secondary metabolism, and we observed that high rates of the regulatory genes in these bacteria control the large number of metabolites expressed under different growth conditions (24). Due to the great range of substrates for energy production, bacteria with a large genome size can survive in diverse habitats (24). COG and KEGG function analyses of genomes revealed that genes related to carbohydrate and amino acid metabolism were enriched in PMA *Clostridium* strains (Fig. 3A and B), suggesting that this could help them utilize carbohydrates and amino acids more effectively. Energy metabolism-related genes were important regulatory elements of the bacterial response to environmental stress (39). The pathways of ABC transporters, two-component system, flagellar assembly, and bacterial chemotaxis were also enriched in PMA *Clostridium* strains, most of which were related to environmental adaptation. The two-component system was the main form of bacterial signal transduction, which could regulate the ability of bacteria to perceive and respond to the external environment (23). Bacterial chemotaxis could help the motile bacteria respond to the chemical concentration gradient of substances, making the bacteria tend to beneficial stimuli and avoid harmful stimuli (40). These functions were enriched in PMA *Clostridium* strains, indicating these genes play key roles in the adaptation of PMA *Clostridium* strains to pit mud environment.

Large amounts of lactic acid and ethanol were produced due to the *Lactobacillaceae* and *Kazachstania* dominating the fermentation process of strong-flavored baijiu, and their relative abundances can reach up to 90% (3). High concentrations of lactic acid and ethanol became the growth inhibitors for microorganisms during the fermentation process of strong-flavor baijiu (41, 42). Previous studies showed that during the fermentation of strong-flavor baijiu, the pH of fermented grains could be lower than 4.0, and the pH of pit mud contacted with fermentation grains was 5.87 (26). It is a challenge for most microorganisms to survive in this acidic environment (43). Pan-GWAS analysis showed that the proportion of PMA *Clostridium* strains with the gene encoding alcohol dehydrogenase and l-lactate dehydrogenase was significantly higher than that of NPMA *Clostridium* strains, and the enrichment is beneficial for the PMA *Clostridium* strains to counteract the stress of lactic acid and ethanol in the fermentation environment. In previous work, good acid resistance and the potential of ethanol and lactic acid metabolism of a *Bacillus* strain and a *Clostridium* strain isolated from strong-flavor baijiu fermentation environment were detected through the analysis of genomic and phenotypic features (44, 45). Further, the enrichment of PMA *Clostridium* strains of the genes involved in citrate cycle may contribute to the metabolism of organic acids and the survival of in the organic acid-rich environment of pit mud (3).

There were significant differences in the type and amount of the genes associated with carbohydrate utilization among *Clostridium* strains in different habitats (46). GHs were the most abundant family of CAZymes in PMA *Clostridium* strains. GHs were always produced by bacteria to degrade polysaccharides to release the key resources for their growth (47, 48). The genes encoding GTs were enriched in PMA *Clostridium* strains. Exopolysaccharides could be synthesized through the family of GTs, which was conducive to the formation of biofilm by bacteria (49). Moreover, GTs were the key enzymes in the process of glycosylation, which was important for the formation of bacterial cell walls (49). The formation of biofilm and cell walls contributed to the resistance of bacteria to environmental stress (50). Therefore, GTs play key roles in the adaptation to the environment of bacteria. GH13 was observed in most bacteria that belonged to the families targeting starch and glycogen (30). GH4 is involved in starch and sucrose metabolism (51). Since starch is the most abundant carbohydrate in pit mud, the enrichment of the genes encoding GH13 and GH4 was helpful for the utilization of resources by PMA *Clostridium* strains in pit mud.

Horizontal gene transfer plays a crucial role in the evolution of bacterial genomes (14, 31). The horizontally transferred genes among PMA strains were similar to those in recent studies, where the most frequent functions of the HGT genes belonged to COG categories C, G, and J (32, 52). Further, these functions (including C, G, E, etc.) were enriched in PMA *Clostridium* strains, indicating HGTs contributed to the functionality of PMA *Clostridium* strains. In this study, a large number of genes associated with carbohydrate and amino acid metabolism were transferred from *Bacillus* to *Clostridium*, which was consistent with a previous study that found bacteria could share nutrients in the environment by transferring genes related to metabolism (31). Multiple genes encoding GH4, GH13, and GH135 were transferred from *Bacillus* to *Clostridium*. GH13 can be directly involved in the metabolism of glycogen and starch (30). GH4 and GH13 were enriched in PMA *Clostridium* strains, indicating that HGTs increased the genes encoding the enzymes for carbohydrate utilization in PMA *Clostridium* strains, which was conducive to the utilization of resources by PMA *Clostridium* strains.

The short-chain fatty acids produced by *Clostridium* are important flavor compounds in Chinese strong-flavor baijiu (7–9). Butyric acid is synthesized via a carboxylic acid chain elongation process that employs reverse oxidation of acetic acid as an electron donor (53, 54). Our results show that the number of genes encoding the enzymes (alcohol dehydrogenase and l-lactate dehydrogenase) that metabolize ethanol and lactic acid directly in PMA *Clostridium* strains was significantly higher than that of NPMA *Clostridium* strains. Not only being easily converted to acetyl-CoA, the oxidation of ethanol and lactic acid offers a number of reducing equivalents that can support medium-chain carboxylic acid synthesis (55). The good potential of PMA *Clostridium* strains to synthesize short-chain fatty acids from ethanol and lactic acid can not only produce key flavor compounds for strong-flavor Baijiu but also alleviate the stresses of ethanol and lactic acid in pit mud (45). Short-chain fatty acids produced by *Clostridium* metabolism also can be used as stress factors for other microorganisms to improve their competitiveness in niches (56). Although there were significant differences in the proportion of *Clostridium* strains with a complete butyric acid synthesis pathway between PMA and NPMA *Clostridium* strains, in practice, we should not ignore the interaction of microorganisms because it can significantly affect the growth and metabolism of microorganisms (57).

In conclusion, the genetic basis of PMA *Clostridium* strains’ habitat adaptation and metabolic profiles was investigated. Many genes related to environmental adaptation, including two-component system, flagellar assembly, and bacterial chemotaxis, were enriched in PMA *Clostridium* strains. Genes related to ethanol, organic acid, and carbohydrate metabolism were enriched in PMA *Clostridium* strains that not only contributed to their dominance in the nutrient complex environment but also ensured their great advantage in utilizing the substances enriched in pit mud. PMA *Clostridium* strains showed good potential to produce short-chain fatty acids from starch, lactic acid, and ethanol. Furthermore, it should be noted that *Clostridium* species are highly heterogeneous, and consequently, the species-level diversity of strains in the pit mud and non-pit mud groups might have a certain impact on their comparison results. On the basis of this study, expanding the species pool of *Clostridium* isolated from pit mud will help to improve the understanding of their habitat adaptability and metabolic profiles.

## MATERIALS AND METHODS

### Collection of data sets for comparative genomic analysis.

In this study, 21 *Clostridium* strains were isolated from the pit mud of strong-flavor baijiu fermentation cellars according to our previous work (58). According to the species taxonomic information in the EzBioCloud database (10), 109 genomes of *Clostridium* strains were downloaded from the NCBI-GenBank database (https://www.ncbi.nlm.nih.gov/; data collected in December 2019), including four type and five nontype strains isolated from pit mud and 100 type strains isolated from non-pit mud habitats (e.g., soil and feces). The detailed information of these 130 *Clostridium* strains used for comparative genomic analysis is shown in Table S1 in the supplemental material.

The genomic DNA of 21 *Clostridium* strains isolated from pit mud was extracted using a bacterial genomic DNA isolation kit (GK1072; Generay). The qualified DNA was sent to Beijing Genomics Institute (Shenzhen, China) for whole-genome sequencing using an Illumina HiSeq 4000 system (Illumina, San Diego, CA). Briefly, DNA was sheared randomly by a Bioruptor ultrasonicator (Diagenode, Denville, NJ) to construct libraries for paired-end (2 × 150 bp) sequencing. FastQC v0.11.9 (https://www.bioinformatics.babraham.ac.uk/projects/fastqc/) was used to check the quality of raw reads. To obtain clean reads, raw reads were trimmed by Trimmomatic v0.39 (http://www.usadellab.org/cms/?page=trimmomatic) to filter out the low-quality reads (59). Clean reads were used for genome assembly using SOAPdenovo v1.05 software (60).

Average nucleotide identity (ANI) based on BLAST (ANIb) was calculated using PYANI v0.2.11 to compare the similarity between all of the *Clostridium* strain genomes shown in Table S1 (61). A heat map was created for the visualization of ANIb values (Fig. S2). A genome pair was considered redundant when the ANIb was greater than 99.9%, and in such cases, one of the two genomes was selected randomly and kept in the data set. Finally, 130 nonredundant *Clostridium* genomes were obtained for further analysis. OrthoFinder v2.3.7 was used to identify the orthogroups of all *Clostridium* strains (Table S1) with default parameters, and the pan-, core, and species-specific gene families were identified (62). The amino acid sequences of 304 single-copy core genes then were extracted from the OrthoFinder output files and aligned using MAFFT v7.474 (63). A maximum-likelihood phylogenetic tree was constructed based on the single-copy core genome using Fasttree v2.1.9 (64), which was visualized via iTOL (https://itol.embl.de/) (65).

10.1128/msystems.00297-22.2 FIG S2 Heatmap based on average nucleotide identity (ANIb) between genomes of all *Clostridium* strains, and dendrogram on the left was based on reciprocal pairwise comparison clustering calculated using pyani, a python module, and the names of *Clostridium* isolated from pit mud are highlighted in orange. Download FIG S2, PDF file, 1.1 MB. Copyright © 2022 Fang et al. 2022 Fang et al. https://creativecommons.org/licenses/by/4.0/ This content is distributed under the terms of the Creative Commons Attribution 4.0 International license.

### Genome function and functional enrichment analysis.

Open reading frames (ORFs) of the genomes were predicted using Prokka v1.14.6 (66). The amino acid sequences of these putative ORFs were aligned against Cluster of Orthologous Groups (COG; version 2014-11-10) and Kyoto Encyclopedia of Genes and Genomes (KEGG; version 89.1) databases to obtain their corresponding annotations with an E value threshold of <1e−3, using Diamond v0.9.24 based on similarity searches (67). The pan-genome-wide association studies (pan-GWAS) analysis was performed for identification of habitat-enriched KEGG orthogroups (KOs) using Scoary v1.6.16 depending on the KO presence/absence data set (68). The metabolic pathways of short-chain fatty acids from glucose, ethanol, lactic acid, and amino acids were analyzed based on KEGG database. To identify the genes encoding carbohydrate-active enzymes (CAZymes), the putative ORFs were aligned against the CAZy database (v7) using dbCAN2 and filtered with the default parameters for bacteria (69). Mann-Whitney *U* test (cutoff, *P < *0.05) was performed for pairwise functional comparison between PMA and NPMA strains based on the gene copy numbers of COG and CAZyme categories. PhyloGLM test was used to infer the enrichment and depletion of KEGG pathways and CAZyme-encoding genes through the R package phylolm v2.6.2 (70). Phylogenetically informed principal component analysis (phylo-PCA) was used to visualize the differences in gene compositions between PMA and NPMA *Clostridium* strains using the R package phytools v0.7-80 (71).

### Identification of HGT among pit mud-associated bacteria.

Horizontal gene transfer (HGT) is conducive to the rapid adaptation of bacteria to unstable environments through acquiring novel functions, and it was identified as the main factor for bacteria to adapt to the human-managed environments, including food systems (31, 72). Therefore, we collected the genomes of all pit mud-associated strains reported so far for HGT analysis, including 30 *Clostridium* strains shown in Table S1, 18 non-*Clostridium* bacterial genomes downloaded from NCBI-GenBank database by searching the keyword “pit mud,” and eight non-*Clostridium* bacterial strains isolated from pit mud and sequenced in this study (Table S3). MetaChip v1.10.0 was used to analyze the HGTs among the 56 PMA strains at the phylum, class, order, family, genus, and species levels with default parameters (73). The candidate HGT genes were identified among defined taxonomic groups using a best-hit approach, and the candidate genes were assessed according to the phylogenetic analysis and reconciliation of gene and species trees. The HGTs detected at all classification levels were merged and the replicated HGTs filtered out. The results of HGTs were visualized using gephi v0.9.2 (74).

### Data availability.

The raw genome sequencing results of 30 strains isolated from pit mud in this study have been deposited in the Genome Sequence Archive in National Genomics Data Center (https://bigd.big.ac.cn/gsub/) (75), China National Center for Bioinformation/Beijing Institute of Genomics, Chinese Academy of Sciences, under the accession number CRA005913.
