# Supplementary figures and images for: Comparative Genomics Unveils the Habitat Adaptation and Metabolic Profiles of Clostridium in an Artificial Ecosystem for Liquor Production
Source: mSystems. 2022 May 2;7(3):e00297-22. doi: 10.1128/msystems.00297-22 (PMC9238394; doi:10.1128/msystems.00297-22)

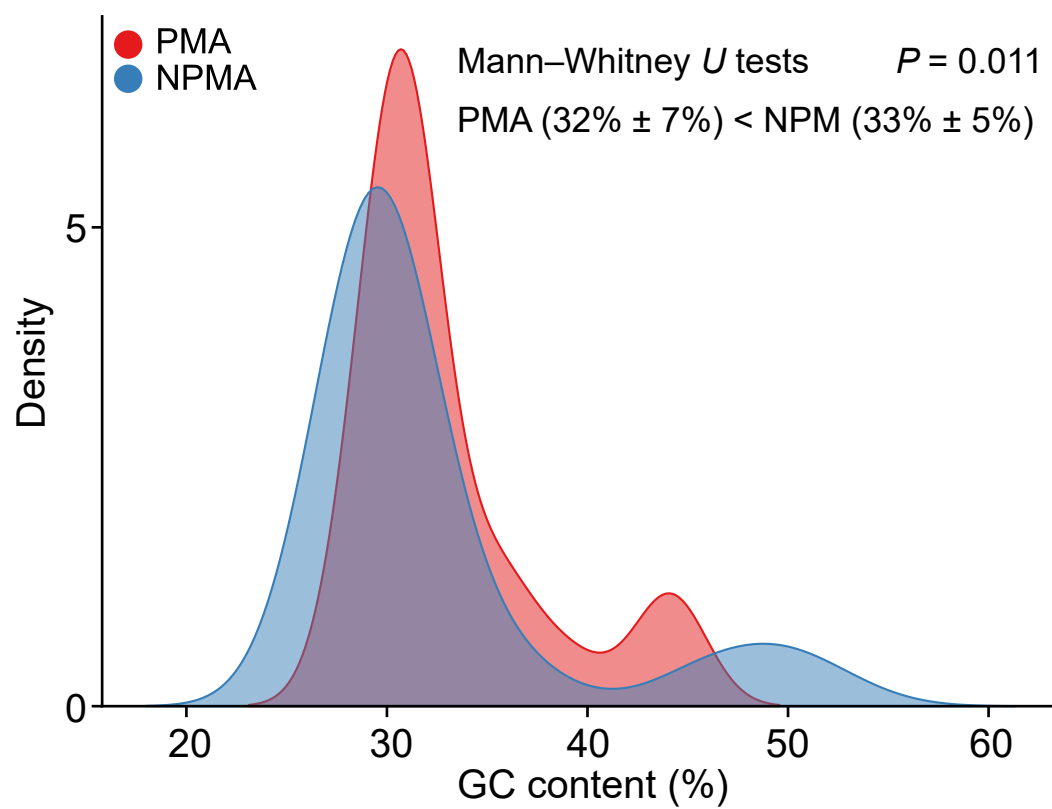

Supplement: FIG S1 [file msystems.00297-22-s0004.pdf]

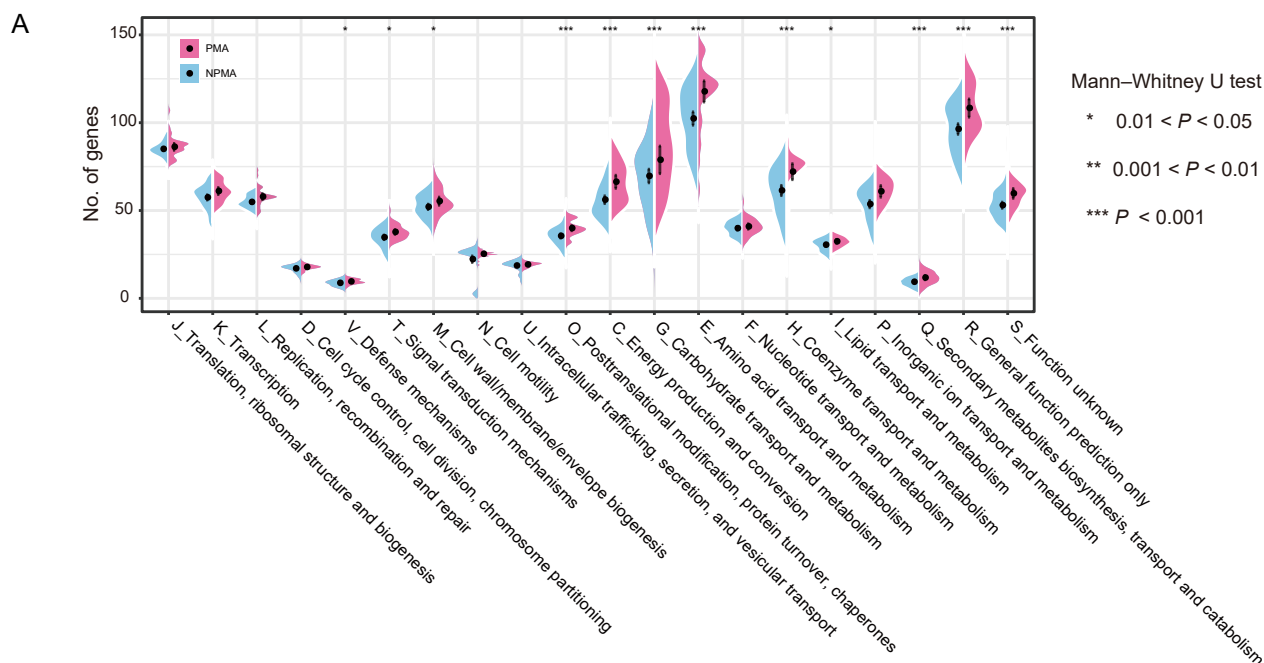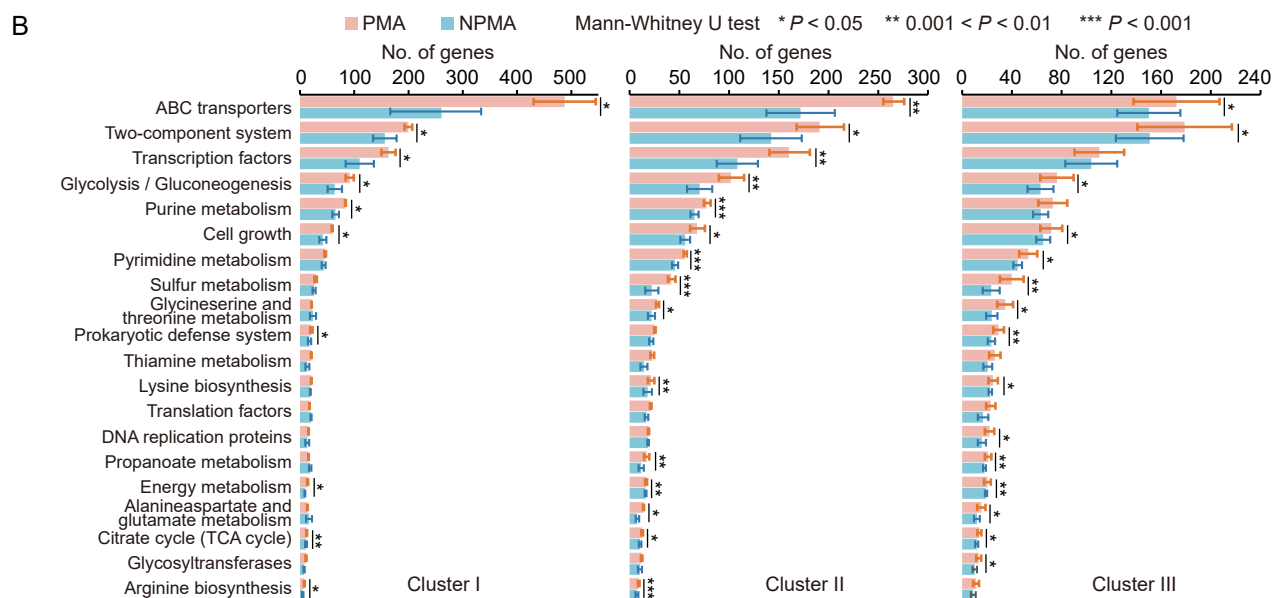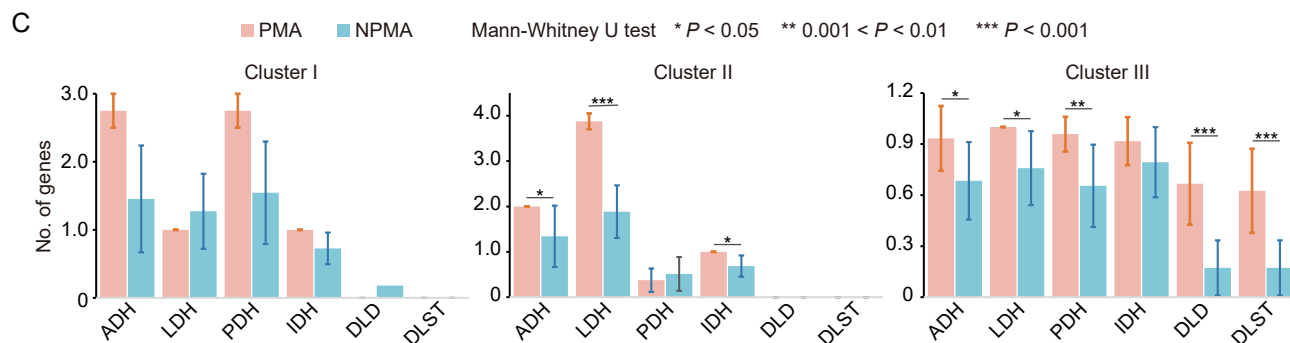

Supplement: FIG S3 [file msystems.00297-22-s0006.pdf]

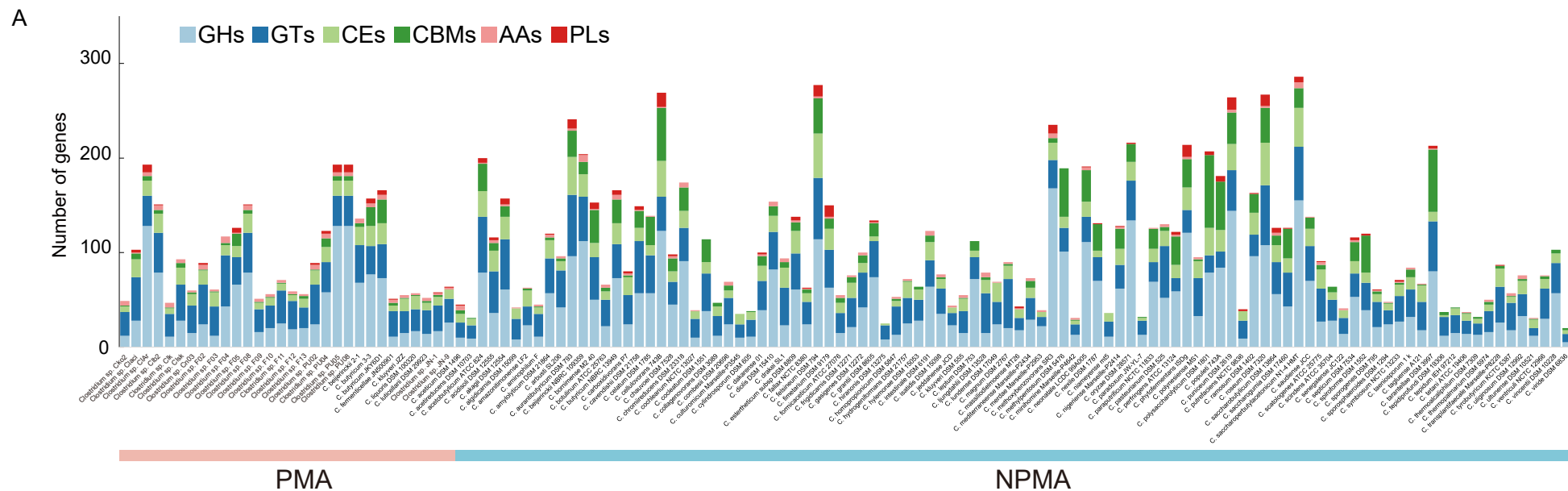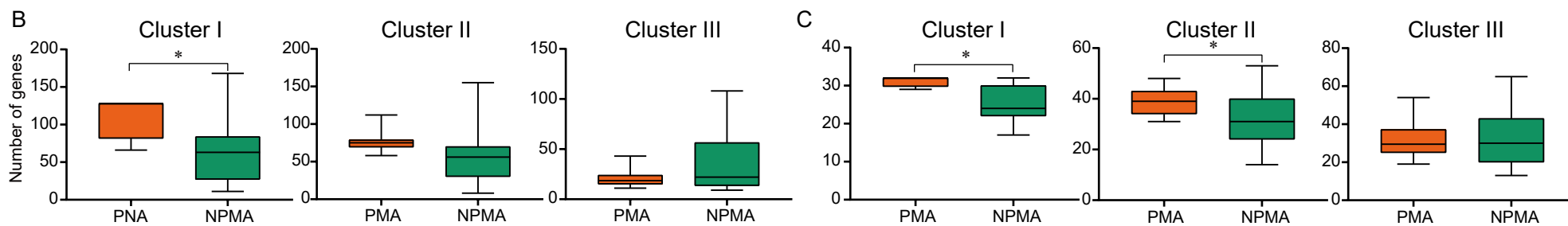

Supplement: FIG S4 [file msystems.00297-22-s0007.pdf]
