# Supplementary material for: Comparative Genomics Unveils the Habitat Adaptation and Metabolic Profiles of Clostridium in an Artificial Ecosystem for Liquor Production
Source: mSystems. 2022 May 2;7(3):e00297-22. doi: 10.1128/msystems.00297-22 (PMC9238394; doi:10.1128/msystems.00297-22)
Supplement: TABLE S1 [file msystems.00297-22-s0001.docx]

| **Taxon name** | **Strain name** | **Accession number** | **Source** | **No. of contigs** | **Genome size (bp)** | **GC content (%)** |
| --- | --- | --- | --- | --- | --- | --- |
| *Clostridium* sp. Cko2  (*C. luticellarii* 97.35%) | Cko2 | This study | Pit mud | 91 | 4,080,931 | 34.4 |
| *Clostridium* sp. ClAr  (*C. indicum* 99.80%) | ClAr | This study | Pit mud | 38 | 5,248,544 | 43.2 |
| *Clostridium* sp. Claci  (*C. aciditolerans* 99.64%) | Claci | This study | Pit mud | 92 | 5,380,062 | 31.3 |
| *Clostridium* sp. Clb2  (*C. beijerinckii* 99.92%) | Clb2 | This study | Pit mud | 224 | 6,046,938 | 29.7 |
| *Clostridium* sp. Clk  (*C. luticellarii* 97.35%) | Clk | This study | Pit mud | 151 | 3,842,459 | 34.3 |
| *Clostridium* sp. Clsk  (*C. sporogenes* 99.58%) | Clsk | This study | Pit mud | 200 | 6,567,563 | 29.8 |
| *Clostridium* sp. Cn03  (*C. homopropionicum* 96.93%) | Cn03 | This study | Pit mud | 68 | 3,039,868 | 30.6 |
| *Clostridium* sp. F02  (*C. carboxidivorans* 100%) | F02 | This study | Pit mud | 210 | 5,958,797 | 31.1 |
| *Clostridium* sp. F03  (Terrisporobacter glycolicus 99.37%) | F03 | This study | Pit mud | 213 | 4,123,222 | 28.8 |
| *Clostridium* sp. F04  (*C. luticellarii* 97.35%) | F04 | This study | Pit mud | 218 | 6,499,543 | 38.4 |
| *Clostridium* sp. F05  (Anaerocolumna aminovalerica 97.62%) | F05 | This study | Pit mud | 199 | 4,888,606 | 35.7 |
| *Clostridium* sp. F08  (*C. beijerinckii* 99.92%) | F08 | This study | Pit mud | 249 | 6,064,696 | 29.7 |
| *Clostridium* sp. F09  (*C. tyrobutyricum* 100%) | F09 | This study | Pit mud | 85 | 3,078,021 | 30.8 |
| *Clostridium* sp. F10  (*C. tyrobutyricum* 99.51%) | F10 | This study | Pit mud | 72 | 3,046,585 | 30.4 |
| *Clostridium* sp. F11  (*C. tyrobutyricum* 100%) | F11 | This study | Pit mud | 77 | 2,945,017 | 30.7 |
| *Clostridium* sp. F12  (*C. tyrobutyricum* 100%) | F12 | This study | Pit mud | 64 | 3,029,174 | 31.0 |
| *Clostridium* sp. F13  (*C. sporogenes* 99.58%) | F13 | This study | Pit mud | 148 | 3,975,972 | 27.6 |
| *Clostridium* sp. PU02  (*C. carboxidivorans* 100%) | PU02 | This study | Pit mud | 160 | 5,664,815 | 29.9 |
| *Clostridium* sp. PU04  (*C. butyricum* 98.69%) | PU04 | This study | Pit mud | 164 | 4,301,030 | 28.6 |
| *Clostridium* sp. PU05  (*C. indicum* 99.80%) | PU05 | This study | Pit mud | 57 | 5,259,066 | 43.2 |
| *Clostridium* sp. PU08  (*C. indicum* 99.82%) | PU08 | This study | Pit mud | 67 | 5,264,564 | 43.2 |
| *Clostridium fermenticellae* | JN500901(Type) | GCA_003600355.1 | pit mud | 1 | 2,819,963 | 31.0 |
| *Clostridium sp.* | JN-1(Type) | GCA_003718715.1 | pit mud | 1 | 2,795,954 | 30.7 |
| *Clostridium sp* | JN-9(Type) | GCA_004103695.1 | pit mud | 1 | 3,223,191 | 32.3 |
| *Clostridium liquoris* | DSM 100320(Type) | GCA_002995785.1 | pit mud | 75 | 2,876,197 | 31.0 |
| *Clostridium luticellarii* | DSM 29923(Type) | GCA_002995845.1 | pit mud | 165 | 3,754,778 | 35.0 |
| *Clostridium beijerinckii* | 2-1 | GCA_002915295.1 | pit mud | 328 | 5,626,308 | 29.8 |
| *Clostridium kluyveri* | JZZ | GCA_001902295.1 | pit mud | 2 | 4,512,934 | 31.6 |
| *Clostridium butyricum* | JKY6D1 | GCA_001465175.1 | pit mud | 3 | 4,618,327 | 28.7 |
| *Clostridium butyricum* | 3-3 | GCA_002915335.1 | pit mud | 342 | 4,388,540 | 28.8 |
| *Clostridium acetobutylicum* | ATCC 824(Type) | GCA_000008765.1 | Plant-derived foodstuff, cornmeal | 2 | 4,132,880 | 30.9 |
| *Clostridium kluyveri* | DSM 555(Type) | GCA_000016505.1 | Mud of a canal | 2 | 4,023,800 | 32.0 |
| *Clostridium phytofermentans* | ISDg(Type) | GCA_000018685.1 | forest soil | 1 | 4,847,594 | 35.3 |
| ***Clostridium ljungdahlii*** | DSM 13528(Type) | GCA_000143685.1 | chicken yard waste | 1 | 4,630,065 | 31.1 |
| *Clostridium cellulovorans* | 743B(Type) | GCA_000145275.1 | Woody biomass digester | 1 | 5,262,222 | 31.2 |
| ***Clostridium arbusti*** | SL206(Type) | GCA_000246895.2 | pear orchard soil | 243 | 3,970,278 | 29.9 |
| *Clostridium senegalense* | JC122(Type) | GCA_000285575.1 | Clinical (human stool) | 83 | 3,925,888 | 26.5 |
| *Clostridium saccharoperbutylacetonicum* | N1-4(HMT)(Type) | GCA_000340885.1 | soil | 2 | 6,666,445 | 29.5 |
| ***Clostridium saccharogumia*** | DSM 17460(Type) | GCA_000686665.1 | faecal sample of a healthy male adult | 133 | 3,143,441 | 30.1 |
| *Clostridium hydrogeniformans* | DSM 21757(Type) | GCA_000686705.1 | from contaminated groundwater | 31 | 4,101,506 | 29.4 |
| *Clostridium akagii* | DSM 12554(Type) | GCA_000686725.1 | beech litter | 50 | 4,589,511 | 30.7 |
| *Clostridium saccharobutylicum* | DSM 13864(Type) | GCA_000473995.1 | soy beans | 1 | 5,107,814 | 28.7 |
| ***Clostridium bornimense*** | M2/40(Type) | GCA_000577895.1 | mesophilic, two-phase, laboratory-scale biogas reactor | 2 | 3,617,025 | 29.5 |
| *Clostridium sporosphaeroides* | DSM 1294(Type) | GCA_000383295.1 | Infected wound | 21 | 3,174,421 | 53.5 |
| *Clostridium pasteurianum* | DSM 525(Type) | GCA_000807255.1 | soil | 1 | 4,352,101 | 29.9 |
| *Clostridium jeddahense* | JCD(Type) | GCA_000577335.1 | feces from an obese 24-year-old male volunteer | 104 | 3,613,503 | 51.9 |
| *Clostridium saudiense* | JCC(Type) | GCA_000577815.1 | feces from an obese 24-year-old man | 100 | 3,653,762 | 27.9 |
| *Clostridium scatologenes* | ATCC 25775(Type) | GCA_000968375.1 | source unknown | 1 | 5,749,410 | 29.6 |
| ***Clostridium sporogenes*** | DSM 795(Type) | GCA_001020205.1 | soil | 1 | 4,142,990 | 28.0 |
| *Clostridium carboxidivorans* | P7(Type) | GCA_001038625.1 | agricultural settling lagoon | 2 | 5,752,782 | 29.9 |
| ***Clostridium lundense*** | DSM 17049(Type) | GCA_000619945.1 | cow rumen fluid | 52 | 4,753,597 | 29.3 |
| *Clostridium viride* | DSM 6836(Type) | GCA_000620945.1 | anaerobic sewage sludge | 7 | 2,415,478 | 49.3 |
| ***Clostridium celatum*** | DSM 1785(Type) | GCA_000320405.1 | Human faeces | 122 | 3,553,721 | 27.7 |
| *Clostridium methoxybenzovorans* | SR3(Type) | GCA_000421505.1 | anaerobic methanogenic pilot-scale digester fed with olive mill wastewater | 14 | 7,085,377 | 44.5 |
| *Clostridium aceticum* | DSM 1496(Type) | GCA_001042715.1 | Mud | 2 | 4,207,037 | 35.3 |
| ***Clostridium dakarense*** | 01(Type) | GCA_000499525.1 | Human gut | 257 | 3,735,762 | 28.0 |
| *Clostridium leptum* | DSM 753(Type) | GCA_000154345.1 | human faeces | 21 | 3,270,209 | 50.2 |
| *Clostridium spiroforme* | DSM 1552(Type) | GCA_000154805.1 | human faeces | 12 | 2,507,885 | 28.6 |
| *Clostridium nexile* | DSM 1787(Type) | GCA_000156035.2 | human faeces | 99 | 3,995,628 | 38.7 |
| *Clostridium methylpentosum* | DSM 5476(Type) | GCA_000158655.1 | human faeces | 17 | 3,478,423 | 50.7 |
| ***Clostridium polynesiense*** | MS1(Type) | GCA_000820705.1 | human feces from a healthy male | 161 | 3,560,738 | 34.0 |
| *Clostridium cylindrosporum* | DSM 605(Type) | GCA_001047375.1 | soil | 28 | 2,735,032 | 31.4 |
| ***Clostridium homopropionicum*** | DSM 5847(Type) | GCA_001263795.1 | anoxic sewage sludge | 48 | 3,650,535 | 31.1 |
| ***Clostridium niameyense*** | mt5(Type) | GCA_001243045.1 | faecal sample collected from a patient with anorexia | 6 | 2,542,842 | 27.4 |
| *Clostridium botulinum* | ATCC 25763(Type) | GCA_001276985.1 | source unknown | 28 | 3,889,092 | 28.0 |
| *Clostridium paradoxum* | JW-YL-7(Type) | GCA_001584565.1 | sewage treatment aeration pool | 3 | 1,925,591 | 30.2 |
| *Clostridium tepidiprofundi* | DSM 19306(Type) | GCA_001594005.1 | deep-sea hydrothermal vent chimney | 175 | 3,060,113 | 29.4 |
| ***Clostridium magnum*** | DSM 2767(Type) | GCA_001623875.1 | freshwater sediment | 25 | 6,634,930 | 32.1 |
| ***Clostridium algidicarnis*** | DSM LF2. (Type) | jgi.1107655.1 | vacuum-packed refrigerated pork | 42 | 2,835,143 | 30.2 |
| *Clostridium fimetarium* | DSM 9179(Type) | jgi.1085809.1 | cattle manure | 48 | 4,568,183 | 33.6 |
| *Clostridium frigidicarnis* | DSM 12271(Type) | jgi.1068062.1 | acuum-packed beef | 112 | 4,337,414 | 28.4 |
| ***Clostridium gasigenes*** | DSM 12272(Type) | jgi.1068064.1 | acuum-packed chilled lamb | 43 | 4,056,506 | 28.7 |
| ***Clostridium uliginosum*** | DSM 12992(Type) | jgi.1085731.1 | acidic peat bog | 78 | 4,080,971 | 28.1 |
| *Clostridium tyrobutyricum* | KCTC 5387(Type) | GCA_001642655.1 | Freeze-dried stock | 2 | 3,134,437 | 31.0 |
| *Clostridium mediterraneense* | Marseille-P2434(Type) | GCA_900091705.1 | stool sample of a 66-year-old diabetic patient | 5 | 3,083,338 | 27.3 |
| ***Clostridium acetireducens*** | DSM 10703(Type) | GCA_001758365.1 | anaerobic bioreactor fed on waste from potato starch factory | 85 | 2,424,212 | 26.7 |
| ***Clostridium formicaceticum*** | ATCC 27076(Type) | GCA_001854185.1 | Sewage plant | 1 | 4,586,755 | 35.5 |
| *Clostridium grantii* | DSM 8605(Type) | GCA_900129965.1 | mullet gut | 87 | 4,474,501 | 29.3 |
| *Clostridium estertheticum subsp. Estertheticum* | DSM 8809(Type) | GCA_001877035.1 | vacuum packed beef | 2 | 4,785,613 | 30.9 |
| *Clostridium collagenovorans* | DSM 3089(Type) | GCA_900130005.1 | sewage sludge digester | 30 | 3,482,404 | 28.9 |
| *Clostridium amylolyticum* | DSM 21864(Type) | GCA_900142075.1 | H2-producing upflow anaerobic sludge blanket reactor | 20 | 4,262,456 | 32.1 |
| ***Clostridium intestinale*** | DSM 6191(Type) | GCA_900130055.1 | faeces of cattle | 33 | 4,600,598 | 30.0 |
| *Clostridium cavendishii* | DSM 21758(Type) | GCA_900141845.1 | contaminated groundwater | 56 | 4,987,666 | 27.9 |
| *Clostridium nigeriense* | Marseille-P2414(Type) | GCA_900086595.1 | Stool sample of a healthy girl from Niger | 4 | 3,799,489 | 28.0 |
| *Clostridium aurantibutyricum* | DSM 793(Type) | GCA_002006235.1 | source unknown | 221 | 4,922,827 | 29.9 |
| *Clostridium puniceum* | DSM 2619(Type) | GCA_002006345.1 | rotting potatoes | 245 | 6,082,167 | 28.6 |
| ***Clostridium felsineum*** | DSM 794(Type) | GCA_002006355.1 | source unknown | 100 | 5,178,654 | 29.9 |
| *Clostridium roseum* | DSM 7320(Type) | GCA_002006215.1 | German maize | 124 | 5,067,725 | 29.8 |
| *Clostridium aminophilum* | F(Type) | GCA_900112885.1 | bovine rumen | 31 | 3,113,820 | 50.7 |
| *Clostridium thermoalcaliphilum* | DSM 7309(Type) | GCA_002029295.1 | sewage sludge | 40 | 2,025,488 | 31.0 |
| ***Clostridium chromiireducens*** | DSM 23318(Type) | GCA_002029255.1 | chromium-contaminated wetland soil | 188 | 5,447,708 | 30.1 |
| ***Clostridium oryzae*** | DSM 28571(Type) | GCA_002029235.1 | rice field soil | 176 | 5,074,802 | 33.1 |
| *Clostridium tetani* | ATCC 19406(Type) | GCA_900167265.1 | Septic wounds | 33 | 2,791,217 | 28.6 |
| *Clostridium tepidum* | IEH 97212(Type) | GCA_002008345.1 | Bloated non-protein milk bottle | 138 | 3,074,397 | 27.1 |
| *Clostridium acidisoli* | DSM 12555(Type) | GCA_900176305.1 | acidic peat bog | 62 | 3,999,957 | 30.1 |
| *Clostridium merdae* | Marseille-P2953(Type) | GCA_900176635.1 | stool samples of healthy French patients | 4 | 2,748,860 | 45.3 |
| ***Clostridium massiliodielmoense*** | MT26(Type) | GCA_900176615.1 | stool sample of a healthy 28-month-old Senegalese boy | 9 | 2,759,335 | 28.4 |
| *Clostridium polysaccharolyticum* | DSM 1801(Type) | GCA_900111595.1 | sheep rumen | 54 | 3,458,343 | 36.5 |
| *Clostridium populeti* | 743A(Type) | GCA_900112775.1 | woody biomass digestor | 44 | 4,222,819 | 36.0 |
| ***Clostridium cochlearium*** | NCTC 13027(Type) | GCA_900187165.1 | Soil | 1 | 2,435,419 | 28.7 |
| *Clostridium chauvoei* | DSM 7528(Type) | GCA_002327185.1 | bovine blackleg | 1 | 2,872,664 | 28.3 |
| ***Clostridium amazonitimonense*** | LF2(Type) | GCA_000753355.2 | Stool sample of a healthy middle-aged woman from Amazonia | 4 | 3,761,428 | 30.5 |
| *Clostridium combesii* | DSM 20696(Type) | GCA_002749945.1 | Soil | 38 | 3,892,013 | 28.1 |
| *Clostridium taeniosporum* | 1/k(Type) | GCA_001735765.2 | Lake Mainaki silt | 4 | 3,503,506 | 26.6 |
| *Clostridium neonatale* | LCDC 99A005(Type) | GCA_002553615.1 | blood(host:Homo sapiens) | 7 | 4,705,520 | 28.5 |
| *Clostridium minihomine* | Marseille-P4642(Type) | GCA_900240385.1 | stool | 12 | 2,705,425 | 46.1 |
| ***Clostridium vincentii*** | DSM 10228(Type) | GCA_002995745.1 | anaerobic sediment | 100 | 3,505,833 | 30.7 |
| ***Clostridium thermopalmarium*** | DSM 5974(Type) | GCA_002995795.1 | palm wine | 74 | 2,819,317 | 31.0 |
| *Clostridium drakei* | SL1(Type) | GCA_003096175.1 | Environmetal | 1 | 5,695,241 | 29.7 |
| *Clostridium fallax* | NCTC 8380(Type) | GCA_900461065.1 | Soil | 15 | 2,852,282 | 27.1 |
| *Clostridium ventriculi* | NCTC 12966(Type) | GCA_900456775.1 | Soil | 14 | 2,549,638 | 27.7 |
| *Clostridium paraputrificum* | NCTC 11833(Type) | GCA_900447045.1 | source unknown | 20 | 3,627,436 | 30.0 |
| ***Clostridium putrefaciens*** | NCTC 9836(Type) | GCA_900461105.1 | ham | 5 | 3,121,953 | 30.4 |
| *Clostridium isatidis* | DSM 15098(Type) | GCA_002285495.1 | fermenting woad vat | 1 | 2,869,927 | 28.9 |
| *Clostridium septicum* | DSM 7534(Type) | GCA_003606265.1 | source unknown | 2 | 3,404,718 | 27.8 |
| *Clostridium tagluense* | A121(Type) | GCA_003865095.1 | permafrost sample | 168 | 5,195,089 | 30.9 |
| *Clostridium symbiosum* | NCTC 13233(Type) | GCA_900461275.1 | source unknown | 3 | 5,054,777 | 47.8 |
| *Clostridium transplantifaecale* | Marseille-P8228(Type) | GCA_900626075.1 | stool | 10 | 5,650,377 | 48.1 |
| *Clostridium scindens* | ATCC 35704(Type) | GCA_004295125.1 | (host:Homo sapiens) | 1 | 3,658,040 | 46.2 |
| *Clostridium butyricum* | NBRC 13949(Type) | GCA_006742065.1 | Intestine of pig | 4 | 4,705,096 | 28.7 |
| ***Clostridium culturomicum*** | Marseille-P3545(Type) | GCA_902143515.1 | stool | 10 | 3,058,214 | 29.4 |
| *Clostridium beijerinckii* | NBRC 109359(Type) | GCA_007992515.1 | Soil | 257 | 5,660,510 | 29.6 |
| *Clostridium hylemonae* | DSM 15053(Type) | GCA_008281175.1 | Human faeces | 1 | 3,833,740 | 48.9 |
| *Clostridium tarantellae* | DSM 3997(Type) | GCA_009295725.1 | black mullet (Mugil cephalus) brain | 713 | 3,979,886 | 25.8 |
| *Clostridium diolis* | DSM 15410(Type) | GCA_008705175.1 | decaying straw | 1 | 5,940,808 | 29.9 |
| ***Clostridium perfringens*** | ATCC 13124 (Type) | GCA_000013285.1 | source unknown | 1 | 3,260,000 | 28.4 |
| *Clostridium hiranonis* | DSM 13275(Type) | GCA_000156055.1 | Clinical (human faeces) | 26 | 2,479,772 | 30.3 |
| ***Clostridium ultunense*** | DSM 10521(Type) | GCA_000511955.1 | mesophilic triculture | 12 | 3,216,692 | 31.5 |
| *Clostridium cocleatum* | DSM 1551(Type) | GCA_900102365.1 | source unknown | 88 | 2,960,000 | 28.5 |
| *Clostridium ramosum* | DSM 1402(Type) | GCA_000154485.1 | source unknown | 12 | 3,240,000 | 31.4 |

Note: The brackets behind the taxon name of the strains isolated in this study represent the closest species and similarity through 16S rRNA sequence alignment. The NPMA strains with bold taxon names were used for the analysis of core-genome functions.
