# Supplementary material for: Comparative Genomics Unveils the Habitat Adaptation and Metabolic Profiles of Clostridium in an Artificial Ecosystem for Liquor Production
Source: mSystems. 2022 May 2;7(3):e00297-22. doi: 10.1128/msystems.00297-22 (PMC9238394; doi:10.1128/msystems.00297-22)
Supplement: TABLE S2 [file msystems.00297-22-s0002.docx]

| Pathways | Pathway ID | Number of genes | | PhyloGLM estimate |
| --- | --- | --- | --- | --- |
|  |  | PMA | NPMA |  |
| ABC transporters | 02010 | 219.8 ± 80.0 | 172.8 ± 86.9 | 0.004458 |
| Two-component system | 02020 | 168.9 ± 67.3 | 141.1 ± 59.1 | 0.007051 |
| Transcription factors | 03000 | 126.6 ± 44.5 | 100.7 ± 41.8 | 0.013459 |
| Glycolysis / Gluconeogenesis | 00010 | 83.2 ± 26.9 | 63.4 ± 23.6 | 0.029529 |
| Purine metabolism | 00230 | 74.9 ± 19.3 | 62.1 ± 12.0 | 0.06027 |
| Cell growth | 99978 | 68.7 ± 16.6 | 54.9 ± 16.0 | 0.05541 |
| Pyrimidine metabolism | 00240 | 52.8 ± 12.1 | 43.4 ± 7.1 | 0.1037 |
| Sulfur metabolism | 00920 | 36.9 ± 18.0 | 20.11 ± 12.8 | 0.05434 |
| Glycineserine and threonine metabolism | 00260 | 28.6 ± 7.4 | 22.3 ± 6.6 | 0.087994 |
| Prokaryotic defense system | 02048 | 29.9 ± 12.4 | 21.4 ± 9.6 | 0.053645 |
| Thiamine metabolism | 00730 | 24.3 ± 6.5 | 20.8 ± 4.3 | 0.096834 |
| Lysine biosynthesis | 00300 | 24.5 ± 7.9 | 18.5 ± 5.4 | 0.111612 |
| Translation factors | 03012 | 20.1 ± 4.4 | 18.6 ± 1.7 | 0.134354 |
| DNA replication proteins | 03032 | 19.1 ± 5.0 | 16.1 ± 3.5 | 0.132528 |
| Propanoate metabolism | 00640 | 21.8 ± 7.0 | 14.5 ± 7.7 | 0.071107 |
| Energy metabolism | 99982 | 20.6 ± 6.8 | 12.7 ± 6.3 | 0.145721 |
| Alanineaspartate and glutamate metabolism | 00250 | 13.4 ± 2.8 | 10.8 ± 2.7 | 0.264702 |
| Citrate cycle (TCA cycle) | 00020 | 13.8 ± 6.0 | 9.3 ± 4.2 | 0.173066 |
| Glycosyltransferases | 01003 | 12.4 ± 3.8 | 8.8 ± 4.1 | 0.159309 |
| Arginine biosynthesis | 00220 | 11.6 ± 4.2 | 7.8 ± 3.5 | 0.061405 |
| Cationic antimicrobial peptide (CAMP) resistance | 01503 | 10.7 ± 6.4 | 7.9 ± 4.7 | 0.094442 |
| Transcription | 99973 | 9.5 ± 3.8 | 6.4 ± 3.2 | 0.156919 |
| Bacterial chemotaxis | 02030 | 8.1 ± 1.6 | 6.4 ± 3.0 | 0.22854 |
| Carbon fixation pathways in prokaryotes | 00720 | 8.8 ± 6.7 | 5.8 ± 4.48 | 0.097241 |
| Exosome | 04147 | 5.8 ± 2.2 | 4.0 ± 2.0 | 0.31885 |
| Benzoate degradation | 00362 | 5.3 ± 3.8 | 3.3 ± 3.0 | 0.178966 |
| D-Glutamine and D-glutamate metabolism | 00471 | 3.4 ± 0.9 | 3.1 ± 0.3 | 0.65486 |
| Carbohydrate metabolism | 99981 | 4.5 ± 2.7 | 2.6 ± 1.9 | 0.32087 |
| Fatty acid degradation | 00071 | 3.4 ± 2.2 | 2.4 ± 2.0 | 0.199123 |
| Tyrosine metabolism | 00350 | 3.7 ± 1.8 | 2.0 ± 1.6 | 0.42522 |
| Protein processing in endoplasmic reticulum | 04141 | 2.6 ± 0.9 | 2.1 ± 1.1 | 0.42598 |
| Cytoskeleton proteins | 04812 | 2.0 ± 1.8 | 1.3 ± 1.3 | 0.30641 |
| Lipoarabinomannan (LAM) biosynthesis | 00571 | 2.0 ± 1.2 | 1.2 ± 1.1 | 0.45512 |
| Valineleucine and isoleucine degradation | 00280 | 1.9 ± 1.3 | 1.2 ± 1.2 | 0.46252 |
| Lipoic acid metabolism | 00785 | 2.2 ± 2.0 | 0.9 ± 0.7 | 0.49387 |
| Neuroactive ligand-receptor interaction | 04080 | 0.4 ± 0.4 | 0.89 ± 0.5 | -1.36684 |
| Biosynthesis of ansamycins | 01051 | 0.4 ± 0.3 | 0.7 ± 0.5 | -1.13594 |
| Proteasome | 03050 | 0.9 ± 0.8 | 0.4 ± 0.4 | 0.81618 |
| Carbapenem biosynthesis | 00332 | 0.6 ± 0.5 | 0.4 ± 0.4 | 0.74823 |
| Betalain biosynthesis | 00965 | 0.6 ± 0.4 | 0.4 ± 0.3 | 0.81437 |
| Prenyltransferases | 01006 | 0.8 ± 0.6 | 0.3 ± 0.3 | 1.5825 |
| African trypanosomiasis | 05143 | 0.5 ± 0.3 | 0.3 ± 0.1 | 0.75629 |
| Amoebiasis | 05146 | 0.4 ± 0.4 | 0.1 ± 0.1 | 0.93511 |
| Metabolism of xenobiotics by cytochrome P450 | 00980 | 0.3 ± 0.3 | 0.1 ± 0.1 | 1.7434 |
| Monobactam biosynthesis | 00261 | 0.1 ± 0.1 | 0.1 ± 0.1 | 1.59058 |

Note: the number of genes is presented as means ± standard deviations
