# Supplementary material for: Comparative Genomics Unveils the Habitat Adaptation and Metabolic Profiles of Clostridium in an Artificial Ecosystem for Liquor Production
Source: mSystems. 2022 May 2;7(3):e00297-22. doi: 10.1128/msystems.00297-22 (PMC9238394; doi:10.1128/msystems.00297-22)
Supplement: TABLE S3 [file msystems.00297-22-s0003.docx]

| **Organism name** | **Strain name** | **Accession number** | **No. of contigs** | **Genome size (bp)** |
| --- | --- | --- | --- | --- |
| *Aminipila* sp. JN-18 | JN-18 | GCA_004103735.1 | 1 | 2,750,839 |
| *Sporosarcina* sp. REN12 | REN12 | GCF_016820585.1 | 49 | 3,645,214 |
| *Bacillus altitudinis* | G25-132-1 | GCA_015846075.1 | 16 | 3,738,909 |
| *Bacillus licheniformis* | MT-B06 | GCA_003606405.1 | 2 | 4,490,546 |
| *Bacillus renqingensis* | REN2 | GCA_016464375.1 | 143 | 4,584,182 |
| *Bacillus* sp*.* REN10 | REN10 | GCA_016820555.1 | 53 | 3,459,634 |
| *Bacillus vini* | JCM 19841 | GCA_016772275.1 | 1 | 4,309,805 |
| *Bacillus* sp*.*REN3 | REN3 | GCA_017912555.1 | 100 | 4,357,126 |
| *Caproiciproducens sp.* NJN-50 | NJN-50 | GCA_004103755.1 | 1 | 3,308,050 |
| *Clostridium beijerinckii* | 2-1 | GCA_002915295.1 | 328 | 5,626,308 |
| *Clostridium butyricum* | 3-3 | GCA_002915335.1 | 342 | 4,388,540 |
| *Clostridium butyricum* | JKY6D1 | GCA_001465175.1 | 3 | 4,618,327 |
| *Clostridium kluyveri* | JZZ | GCA_001902295.1 | 2 | 4,512,934 |
| *Clostridium liquoris* | DSM 100320(Type) | GCA_002995785.1 | 75 | 2,876,197 |
| *Clostridium luticellarii* | DSM 29923(Type) | GCA_002995845.1 | 165 | 3,754,778 |
| *Clostridium* sp. | JN-1(Type) | GCA_003718715.1 | 1 | 2,795,954 |
| *Clostridium* sp. | JN-9(Type) | GCA_004103695.1 | 1 | 3,223,191 |
| *Devosia* sp. I507 | I507 | GCA_002949085.1 | 1 | 4,005,916 |
| *Lactiplantibacillus plantarum* | C410L1 | GCF_001874125.1 | 7 | 3,392,777 |
| *Pediococcus acidilactici* | JKY18 | GCA_001868705.1 | 3 | 1,878,955 |
| *Planomicrobium* sp. REN14 | REN14 | GCA_016820615.1 | 21 | 3,713,775 |
| *Planomicrobium* sp. REN8 | REB8 | GCA_016820495.1 | 32 | 3,214,327 |
| *Rummeliibacillus suwonensis* | 3B-1 | GCA_017578305.1 | 83 | 4,117,671 |
| *Sphingomonas* sp. REN5 | REN5 | GCA_016820445.1 | 25 | 3,621,233 |
| *Sporosarcina* sp. REN13 | REN13 | GCA_016820535.1 | 83 | 4,231,192 |
| *Tissierellia* sp. JN-28 | JN-28 | GCA_004103715.1 | 1 | 3,743,723 |
| *Anaerocolumna* sp. Ami01  (*Anaerocolumna aminovalerica* 97.62%) | Ami01 | This study | 102 | 4,825,733 |
| *Clostridium* sp. Cko2  (*C. luticellarii* 97.35%) | Cko2 | This study | 91 | 4,080,931 |
| *Clostridium* sp. Claci  (*C. aciditolerans* 99.64%) | Claci | This study | 92 | 5,380,062 |
| *Clostridium* sp. ClAr  (*C. indicum* 99.80%) | ClAr | This study | 38 | 5,248,544 |
| *Clostridium* sp. Clb2  (*C. beijerinckii* 99.92%) | Clb2 | This study | 224 | 6,046,938 |
| *Clostridium* sp. Clk  (*C. luticellarii* 97.35%) | Clk | This study | 151 | 3,842,459 |
| *Clostridium* sp. Cn03  (*C. homopropionicum* 96.93%) | Cn03 | This study | 68 | 3,039,868 |
| *Cutibacterium* sp. Cua  (*Cutibacterium acnes subsp. Defendens* 100%) | Cua | This study | 14 | 2,489,484 |
| *Clostridium* sp. F01  (*Staphylococcus warneri* 99.91%) | F01 | This study | 1498 | 7,111,456 |
| *Clostridium* sp. F02  (*C. carboxidivorans* 100%) | F02 | This study | 210 | 5,958,797 |
| *Clostridium* sp. F03 | F03 | This study | 213 | 4,123,222 |
| *Clostridium* sp. F04  (*C. luticellarii* 97.35%) | F04 | This study | 218 | 6,499,543 |
| *Clostridium* sp. F05 | F05 | This study | 199 | 4,888,606 |
| *Clostridium* sp. F06  (*Clostridium tyrobutyricum* 99.93%) | F06 | This study | 117 | 3,011,985 |
| *Clostridium* sp. F08  (*C. beijerinckii* 99.92%) | F08 | This study | 249 | 6,064,696 |
| *Clostridium* sp. F09  (*C. tyrobutyricum* 100%) | F09 | This study | 94 | 3,104,304 |
| *Clostridium* sp. F10  (*C. tyrobutyricum* 99.52%) | F10 | This study | 118 | 3,058,270 |
| *Clostridium* sp*.* F11  (*C. tyrobutyricum* 100%) | F11 | This study | 144 | 4,770,424 |
| *Clostridium* sp. F12  (*C. tyrobutyricum* 100%) | F12 | This study | 119 | 3,085,917 |
| *Clostridium* sp. F13  (*C. sporogenes* 99.58%) | F13 | This study | 148 | 3,975,972 |
| *Muricomes* sp. Mui05  (*Muricomes intestine* 99.79%) | Mui05 | This study | 166 | 3,892,169 |
| *Clostridium* sp. PU02  (*C. carboxidivorans* 100%) | PU02 | This study | 160 | 5,664,815 |
| *Lactiplantibacillus* sp. PU03  (*Lactiplantibacillus paraplantarum* 100%) | PU03 | This study | 268 | 3,447,401 |
| *Clostridium* sp. PU04  (*C. butyricum* 98.69%) | PU04 | This study | 164 | 4,301,030 |
| *Clostridium* sp. PU05  (*C. indicum* 99.80%) | PU05 | This study | 57 | 5,259,066 |
| *Hungatella* sp*.* PU06 | PU06 | This study | 61 | 5,262,750 |
| *Lactiplantibacillus* sp. PU07  (*Lactiplantibacillus paraplantarum* 100%) | PU07 | This study | 3901 | 6,843,979 |
| *Clostridium* sp*.* PU08  (*C. indicum* 99.82%) | PU08 | This study | 67 | 5,264,564 |
| *Syntrophococcus* sp. Rum  (*Syntrophococcus sucromutans* 92.71%) | Rum | This study | 60 | 3,390,801 |

Note: The brackets behind the taxon name of the strains isolated in this study represent the closest species and similarity through 16S rRNA sequence alignment.
