# Supplementary material for: Comparative Genomics Unveils the Habitat Adaptation and Metabolic Profiles of Clostridium in an Artificial Ecosystem for Liquor Production
Source: mSystems. 2022 May 2;7(3):e00297-22. doi: 10.1128/msystems.00297-22 (PMC9238394; doi:10.1128/msystems.00297-22)
Supplement: FIG S2 [file msystems.00297-22-s0005.pdf]

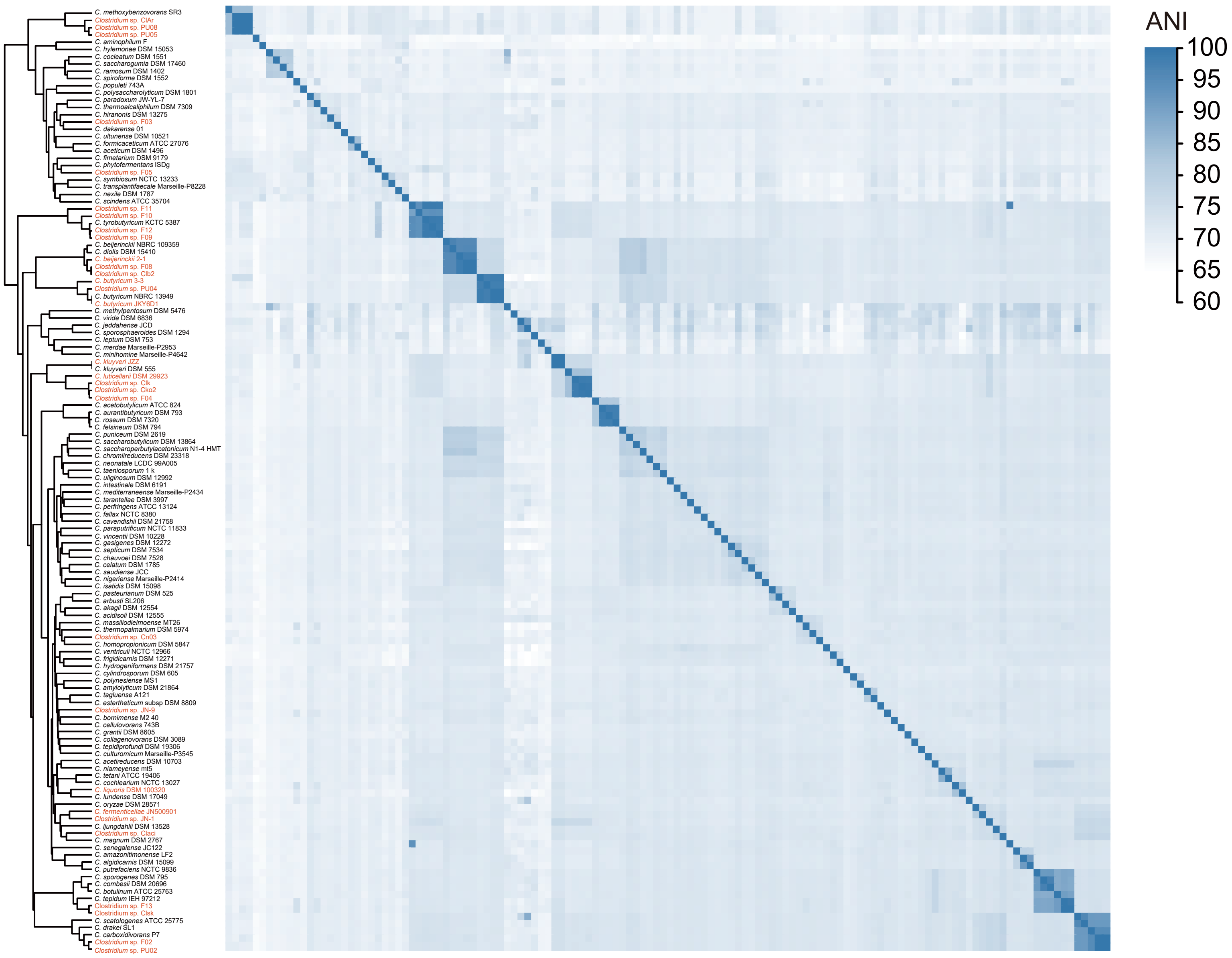

C. methoxybenzovorans SR3
Clostridium sp. ClAr
Clostridium sp. PU08
Clostridium sp. PU05
C. aminophilum F
C. hylemonae DSM 15053
C. cocleatum DSM 1551
C. saccharogumia DSM 17460
C. ramosum DSM 1402
C. spiroforme DSM 1552
C. populeti 743A
C. polysaccharolyticum DSM 1801
C. paradoxum JW-YL-7
C. thermoalcaliphilum DSM 7309
C. hiranonis DSM 13275
Clostridium sp. F03
C. dakarense 01
C. ultunense DSM 10521
C. formicaceticum ATCC 27076
C. aceticum DSM 1496
C. fimetarium DSM 9179
C. phytofermentans ISDg
Clostridium sp. F05
C. symbiosum NCTC 13233
C. transplantifaecale Marseille-P8228
C. nexile DSM 1787
C. scindens ATCC 35704
Clostridium sp. F11
Clostridium sp. F10
C. tyrobutyricum KCTC 5387
Clostridium sp. F12
Clostridium sp. F09
C. beijerinckii NBRC 109359
C. diolis DSM 15410
C. beijerinckii 2-1
Clostridium sp. F08
Clostridium sp. Clb2
C. butyricum 3-3
Clostridium sp. PU04
C. butyricum NBRC 13949
C. butyricum JKY6D1
C. methylpentosum DSM 5476
C. viride DSM 6836
C. jeddahense JCD
C. sporosphaeroides DSM 1294
C. leptum DSM 753
C. merdae Marseille-P2953
C. minihomine Marseille-P4642
C. kluyveri JZZ
C. kluyveri DSM 555
C. luticellarii DSM 29923
Clostridium sp. Clk
Clostridium sp. Cko2
Clostridium sp. F04
C. acetobutylicum ATCC 824
C. aurantibutyricum DSM 793
C. roseum DSM 7320
C. felsineum DSM 794
C. puniceum DSM 2619
C. saccharobutylicum DSM 13864
C. saccharoperbutylacetonicum N1-4 HMT
C. chromiireducens DSM 23318
C. neonatale LCDC 99A005
C. taeniosporum 1 k
C. uliginosum DSM 12992
C. intestinale DSM 6191
C. mediterraneense Marseille-P2434
C. tarantellae DSM 3997
C. perfringens ATCC 13124
C. fallax NCTC 8380
C. cavendishii DSM 21758
C. paraputrificum NCTC 11833
C. vincentii DSM 10228
C. gasigenes DSM 12272
C. septicum DSM 7534
C. chauvoei DSM 7528
C. celatum DSM 1785
C. saudiense JCC
C. nigeriense Marseille-P2414
C. isatidis DSM 15098
C. pasteurianum DSM 525
C. arbusti SL206
C. akagii DSM 12554
C. acidisoli DSM 12555
C. massiliodielmoense MT26
C. thermopalmarium DSM 5974
Clostridium sp. Cn03
C. homopropionicum DSM 5847
C. ventriculi NCTC 12966
C. frigidicarnis DSM 12271
C. hydrogeniformans DSM 21757
C. cylindrosporum DSM 605
C. polynesiense MS1
C. amylolyticum DSM 21864
C. tagluense A121
C. estertheticum subsp DSM 8809
Clostridium sp. JN-9
C. bornimense M2 40
C. cellulovorans 743B
C. grantii DSM 8605
C. collagenovorans DSM 3089
C. tepidiprofundi DSM 19306
C. culturomicum Marseille-P3545
C. acetireducens DSM 10703
C. niameyense mt5
C. tetani ATCC 19406
C. cochlearium NCTC 13027
C. liquoris DSM 100320
C. lundense DSM 17049
C. oryzae DSM 28571
C. fermenticellae JN500901
Clostridium sp. JN-1
C. ljungdahlii DSM 13528
Clostridium sp. Claci
C. magnum DSM 2767
C. senegalense JC122
C. amazonitimonense LF2
C. algidicarnis DSM 15099
C. putrefaciens NCTC 9836
C. sporogenes DSM 795
C. combesii DSM 20696
C. botulinum ATCC 25763
C. tepidum IEH 97212
Clostridium sp. F13
Clostridium sp. Clsk
C. scatologenes ATCC 25775
C. drakei SL1
C. carboxidivorans P7
Clostridium sp. F02
Clostridium sp. PU02
ANI
100
95
90
85
80
75
70
65
60
