# Supplementary material for: Comparative Genomics Unveils the Habitat Adaptation and Metabolic Profiles of Clostridium in an Artificial Ecosystem for Liquor Production
Source: mSystems. 2022 May 2;7(3):e00297-22. doi: 10.1128/msystems.00297-22 (PMC9238394; doi:10.1128/msystems.00297-22)
Supplement: FIG S5 [file msystems.00297-22-s0008.pdf]

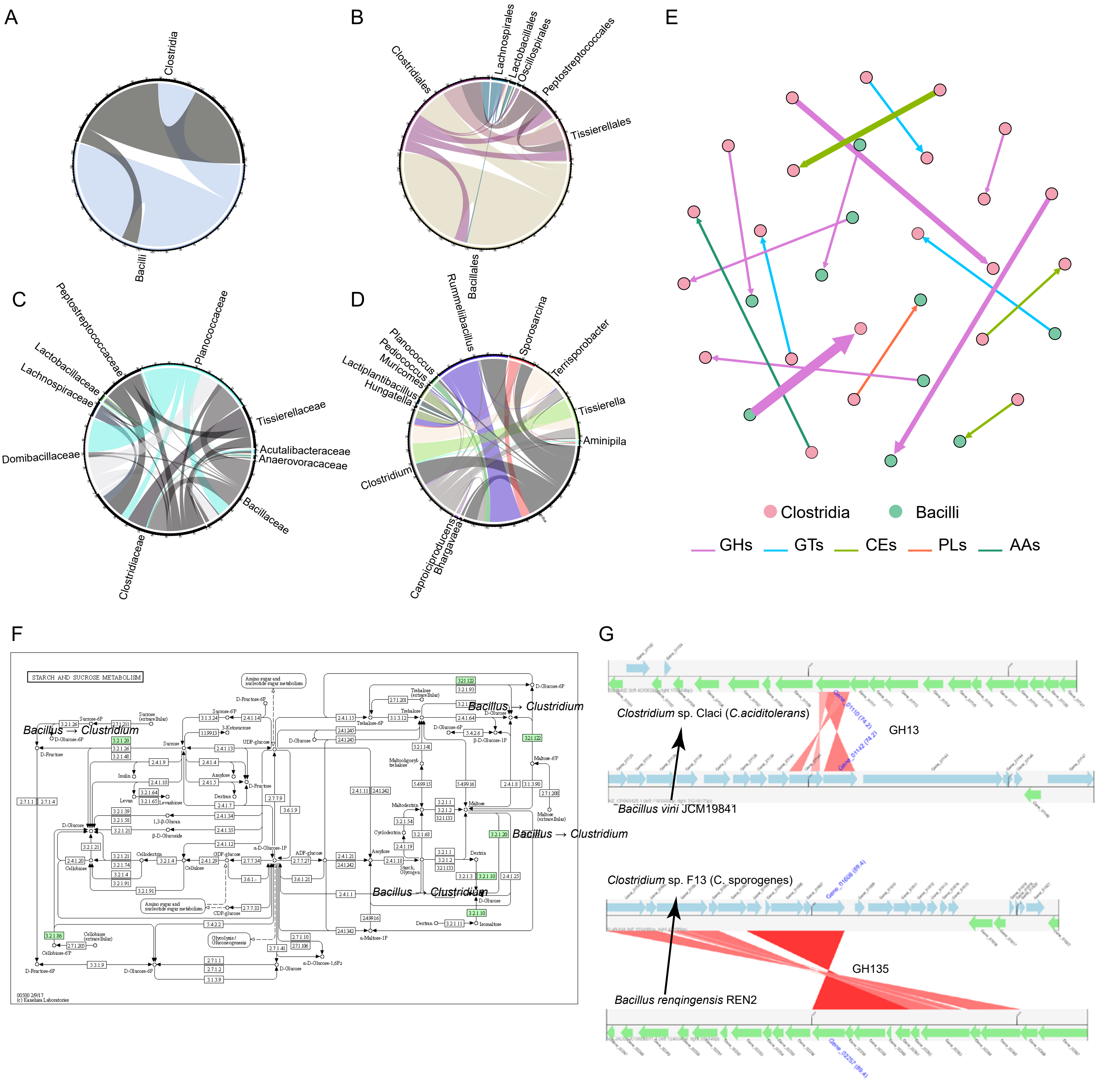

A
Clostridia
Bacilli
B
Clostridiales
Lachnospirales
Lactobacillales
Oscillospirales
Peptostreptococcales
Tissierellales
Bacillales
C
Peptostreptococcaceae
Planococcaceae
Lactobacillaceae
Lachnospiraceae
Tissierellaceae
Acutalibacteraceae
Anaerovoracaceae
Domibacillaceae
Bacillaceae
Clostridiaceae
D
Rummeliibacillus
Sporosarcina
Terrisporobacter
Planococcus
Pediococcus
Muricomes
Lactiplantibacillus
Hungatella
Tissierella
Aminipila
Clostridium
Caproiciproducens
Bhargavaea
E
Clostridia
Bacilli
GHs
GTs
CEs
PLs
AAs
F
STARCH AND SUCROSE METABOLISM
Bacillus → Clustridium
Bacillus → Clustridium
Bacillus → Clustridium
Bacillus → Clustridium
G
Clostridium sp. Claci (C.aciditolerans)
GH13
Bacillus vini JCM19841
Clostridium sp. F13 (C. sporogenes)
GH135
Bacillus renqingensis REN2
